# Stalls in Fertility Transitions in sub‐Saharan Africa: Revisiting the Evidence

**DOI:** 10.1111/sifp.12098

**Published:** 2019-08-05

**Authors:** Bruno Schoumaker

## Abstract

Stalls in fertility decline were first identified in Ghana and Kenya in the early 2000s, and since then as many as 20 African countries have been classified in the “stall” category at some point. The countries and time periods in which they occurred are not well established, however, and whether stalls in sub‐Saharan Africa are pervasive or not remains an open question. This article identifies where and when fertility stalls have occurred in sub‐Saharan Africa. I combine a variety of data sources and methods to identify cases of fertility stalls strongly supported by the data. I find unambiguous support for stalls in two countries (Namibia and Zimbabwe), very strong support in three additional countries (Congo, Kenya, and Zambia), and fairly strong support in Cameroon, in the early 2000s. Stalls are possible in seven cases in six other countries (Côte d'Ivoire, Gabon, Madagascar, Nigeria, South Africa, and Tanzania), where evidence is moderate. Fertility stalls in sub‐Saharan Africa are thus not widespread, but they are not exceptional either. Further research on the causes of these stalls is key to a better understanding of the future paths of fertility in sub‐Saharan Africa.

Fertility transition started much later in sub‐Saharan Africa than in other regions of the world, and the pace of the fertility decline there has been slower overall (Bongaarts 2013; Bongaarts and Casterline 2013; Howse 2015; Shapiro and Hinde 2017). Stalls and reversals in fertility transitions in sub‐Saharan African countries have contributed to the slow declines, and to the uncertainty about sub‐Saharan Africa's future fertility (Schoumaker 2017).

Fertility stalls in sub‐Saharan Africa were first identified in Ghana and Kenya in the early 2000s (Bongaarts 2006). Since then, as many as 20 African countries have been classified in the “stall” category at some point in a dozen or so articles (Westoff and Cross 2006; Agyei‐Mensah 2007; Bongaarts 2008; Garenne 2008, 2011; Moultrie et al. 2008; Shapiro and Gebreselassie 2008; Ezeh, Mberu, and Emina 2009; Schoumaker 2009; Machiyama 2010; Goujon, Lutz, and KC 2015; Sayi 2015; Kebede, Goujon, and Lutz 2019). However, a variety of definitions and methods have been used and, as a result, the list of stalls is far from consistent across authors. For instance, Goujon, Lutz, and KC (2015), using United Nations Population Division data, identified ten stalls in sub‐Saharan Africa, including in countries that were not identified with stalled transitions before (e.g., Congo, The Gambia, Mali, Niger). Their list also did not include Ghana, one of the countries frequently mentioned by other authors (Bongaarts 2006; Agyei‐Mensah 2007; Garenne 2008; Shapiro and Gebreselassie 2008). The quality of some surveys (mainly from the Demographic and Health Surveys program) in the identification of the stalls has also been questioned (Schoumaker 2009, 2014; Machiyama 2010). All in all, the number of stalls and the countries and time periods in which they occurred are not well established, and whether stalls in sub‐Saharan Africa are pervasive or not remains an open question.

The demographic dynamics and the causes of the stalls have also been addressed in several articles, but with mixed results (Bongaarts 2006; Westoff and Cross 2006; Garenne 2008; Moultrie et al. 2008; Shapiro and Gebreselassie 2008; Ezeh, Mberu, and Emina 2009; Sandron 2010; Goujon, Lutz, and KC 2015; Howse, 2015; Kebede, Goujon, and Lutz 2019). While stalls in contraceptive use and in demand for children have been found to be correlated to fertility stalls in Kenya and other Eastern African countries (Westoff and Cross 2006; Ezeh, Mberu, and Emina 2009), this was not found in other research (Shapiro and Gebreselassie 2008; Askew, Maggwa, and Obare 2017). Links between socioeconomic changes and stalls are also not unambiguous. In Kenya, Bongaarts (2006) found that the fertility stall in the late 1990s was accompanied by a leveling off of GDP per capita, of the proportion of women schooled, and of child mortality. In Ghana, he also found a leveling off of the proportion of women schooled and child mortality at the time of the fertility stall, but GDP per capita continued increasing (Bongaarts 2006). Shapiro and Gebreselassie (2008) also obtained mixed results. Slow improvements in education and in child mortality were correlated with fertility stalls; however, slower growth in GDP per capita was associated with more rapid declines in fertility, and trends in GDP per capita could thus not account for stalling fertility.

More recently, Kebede, Goujon, and Lutz (2019) found that stalls in fertility decline in sub‐Saharan Africa could partly be explained by disruptions in the increase of female education. However, as their analysis on Kenya shows, stalls in education do not entirely account for fertility stalls. All in all, these mixed results indicate that further research is needed to better understand these stalls. The results also suggest that measurement errors in fertility may be an issue: some of the stalls included in these studies cannot be accounted for by trends in socioeconomic factors and may not have occurred.

This article identifies where and when fertility stalls have occurred in sub‐Saharan Africa. I combine a variety of data sources and methods to identify cases of fertility stalls strongly supported by the data. As discussed by Gendell (1985) in his early work on fertility stalls, I suggest that further research should focus on these confirmed cases of fertility stalls to reach firmer conclusions about the causes of the stalls.

## DATA AND METHODS

**Figure 1:**
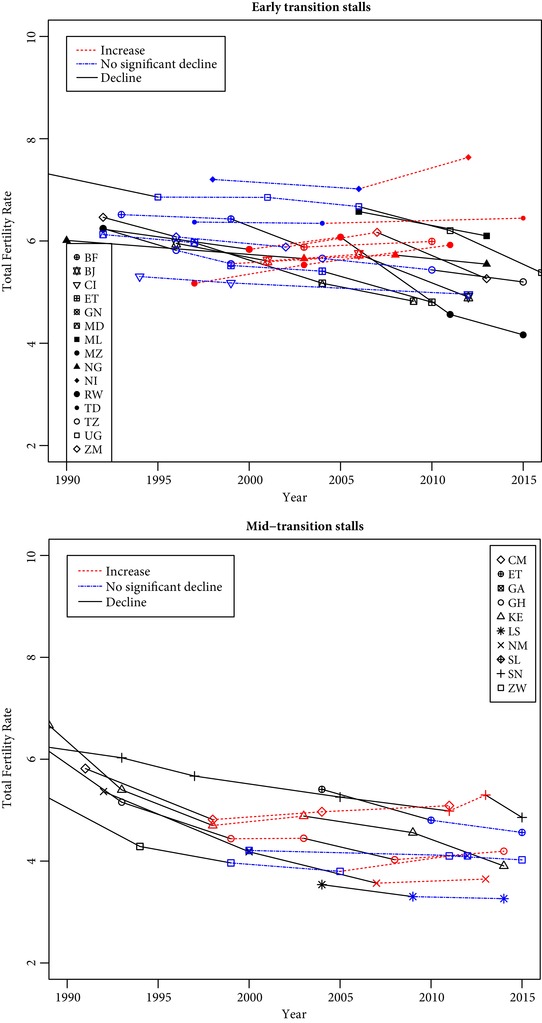
Mid‐transition stalls and early‐transition stalls in sub‐Saharan Africa BF: Burkina Faso, BJ: Benin, CI: Côte d'Ivoire, CM: Cameroon, ET: Ethiopia, GA: Gabon, GH: Ghana, GN: Guinea, KE: Kenya, LS: Lesotho, MD: Madagascar, ML: Mali, MZ: Mozambique, NG: Nigeria, NI: Niger, NM: Namibia, RW: Rwanda, SL: Sierra Leone, SN: Senegal, TD: Chad, TZ: Tanzania, UG: Uganda, ZM: Zambia, ZW: Zimbabwe.

During the last few years, new demographic surveys have been conducted in many sub‐Saharan African countries, covering a larger set of countries and longer time periods than in previous studies on fertility stalls. Census data—which have rarely been used in the analysis of stalls (Garenne et al. 2015)—have also become increasingly available. In the current article, I make use of the following data sources (Table 1):

- Standard Demographic and Health Surveys (DHS) conducted in sub‐Saharan Africa since the 1980s. In total, 125 surveys are used in the 32 countries where at least two surveys have been conducted. Fertility trends are estimated both from published estimates (Figure 1) and with full birth histories.
- Other surveys conducted as part of the DHS program: Malaria Indicator Surveys (MIS), AIDS Indicator Surveys (AIS), DHS interim surveys, DHS special surveys. I treat these surveys separately, as their questionnaires are usually different from the standard DHS. In total, 34 surveys in 18 countries are used. In many countries, only one of these surveys is available and fertility can only be estimated for the few years preceding it. As a result, fertility trends cannot usually be computed from these surveys, but they provide relevant information to evaluate fertility trends in combination with other sources.
- Full birth histories from Multiple Indicator Cluster Surveys (MICS) are available in 11 sub‐Saharan African countries (13 surveys). I use these surveys to reconstruct fertility trends, in the same way as with DHS (details below). Total fertility rates computed from the number of births in the last 12 months are also available in 6 countries (8 surveys).
- I also use census data for the 32 countries. They provide two types of information: (1) total fertility rates computed from the number of births in the last 12 months (in 29 countries), and (2) data on age–sex structure (in 31 countries), used for the reconstruction of fertility trends with the reverse‐survival method (details below). These data are obtained from census reports, the United Nations Statistics Division, and samples of microdata.^1^ In total, data from 86 censuses are used.

**Table 1:** List of countries and data sources

	Data sources, types of methods and indicators, and years of survey				
	Standard DHS and Continuous DHS	MICS	AIS, MIS, Interim DHS, Special DHS	Censuses—age structures	Censuses—births in the last 12 months
	
Country	Published fertility (last three years),reconstructed trends from full birthhistories, expected fertility from theBongaarts model	Reconstructed trends from full birth histories, sometimes published fertility for the last 12 months (in parentheses)	Fertility over the last three years or over the last 12 months, sometimes reconstructed trends from full birth histories	Reverse‐survival methods	Fertility over the last 12 months
Benin	1996, 2001, 2006, 2011–12	2014	—	1992, 2002, 2013	1992, 2002, 2013
Burkina Faso	1993, 1998–99, 2003, 2010	(2006)	2014	1985, 1996, 2006	1985, 1996, 2006
Burundi	1987, 2010, 2016–17	—	2012	1990, 2008	—
Cameroon	1991, 1998, 2004, 2011	2014	—	1987, 2005	1987, 2005
Chad	1996–97, 2004, 2014–15	—	—	2009	1993, 2009
Comoros	1996, 2012	—	—	2003 (5‐y)	1980
Congo	2005, 2011–12	2014–15	2009	1985, 2007	—
Côte d'Ivoire	1994, 1998–99, 2011–12	(2006), 2016	2005	1988, 1998, 2014	1988, 1998, 2014
DR Congo	2007, 2013–14	(2001), (2010)	—	—	1984
Ethiopia	2000, 2005, 2011, 2016	—	—	1994, 2007	1984, 1994, 2007
Gabon	2000, 2012	—	—	1993, 2013 (5‐y)	1993, 2013
Ghana	1988, 1993, 1998, 2003, 2008, 2014	2011	2007, 2016, 2017	2000, 2010	2000, 2010
Guinea	1999, 2005, 2012	2016	—	1983, 1996, 2014	1983, 1996, 2014
Kenya	1989, 1993, 1998, 2003, 2008–09, 2014	—	2015	1989, 1999, 2009	1989, 1999, 2009
Lesotho	2004, 2009, 2014	—	—	2006, 2016 (5‐y)	1976, 1986, 1996, 2006
Liberia	1986, 2007, 2013	—	2009, 2011, 2016	1984, 2008	1984, 2008
Madagascar	1992, 1997, 2003–04, 2008–09	—	2011, 2013, 2016	1993	1975, 1993
Malawi	1992, 2000, 2004, 2010, 2015–16	2006, 2013–14	2012, 2014, 2017	1987, 1998, 2008	1977, 1987, 1998, 2008
Mali	1987, 1995–96, 2001, 2006, 2012–13	2015	2015	1987, 1998, 2009	1987, 2009
Mozambique	1997, 2003, 2011	2008	2015	1980, 1997, 2007	1980, 1997, 2007
Namibia	1992, 2000, 2006–07, 2013	—	—	1991, 2001, 2011	1991, 2001, 2011
Niger	1992, 1998, 2006, 2012	(2000)	—	1988, 2001, 2012	1988, 2001, 2012
Nigeria	1990, 2003, 2008, 2013	(2010), 2016–17	2010, 2015	1991, 2006	—
Rwanda	1992, 2000, 2005, 2010, 2014–15	—	2007–08, 2011, 2013, 2017	1991, 2002, 2012	1978, 1991, 2002, 2012
Senegal	1986, 1992–93, 1997, 2005, 2010–11, 2012–13, 2016	—	2006, 2008–09	1988, 2002, 2013	2002, 2013
Sierra Leone	2008, 2013	(2005), (2010)	2016	2004, 2015 (5‐y)	1974, 1985, 2004, 2015
South Africa	1998, 2016	—	—	1996, 2001, 2011	1996, 2001, 2011
Tanzania	1991–92, 1996, 1999, 2004–05, 2010, 2015–16	—	2007–08, 2011–12, 2017	1988, 2002, 2012	1988, 2002, 2012
Togo	1988, 1998, 2013–14	—	2017	2010 (5‐y)	2010
Uganda	1988–89, 1995, 2000–01, 2006, 2011, 2016	—	2009, 2014–15	1991, 2002, 2014	1991, 2002, 2014
Zambia	1992, 1996, 2001–02, 2007, 2013–14	—	—	1990, 2000, 2010	1990, 2000, 2010
Zimbabwe	1988, 1994, 1999, 2005–06, 2010–11, 2015	2009, 2014	—	1992, 2002, 2012	1992, 2002, 2012

NOTES: Only countries with at least two standard or continuous DHS are included. DHS for which data files are not publicly available are not included (e.g., 2003 South Africa DHS). In a few cases, only five‐year age groups are available in census data (indicated with 5‐y), and trends are reconstructed by five‐year periods. Sub‐Saharan African countries that are not covered are: Angola, Botswana, Cape Verde, Central African Republic, Djibouti, Equatorial Guinea, Eritrea, The Gambia, Guinea Bissau, Mauritania, Mauritius, São Tomé and Príncipe, Somalia, South Sudan, and Swaziland.

**Figure 2:**
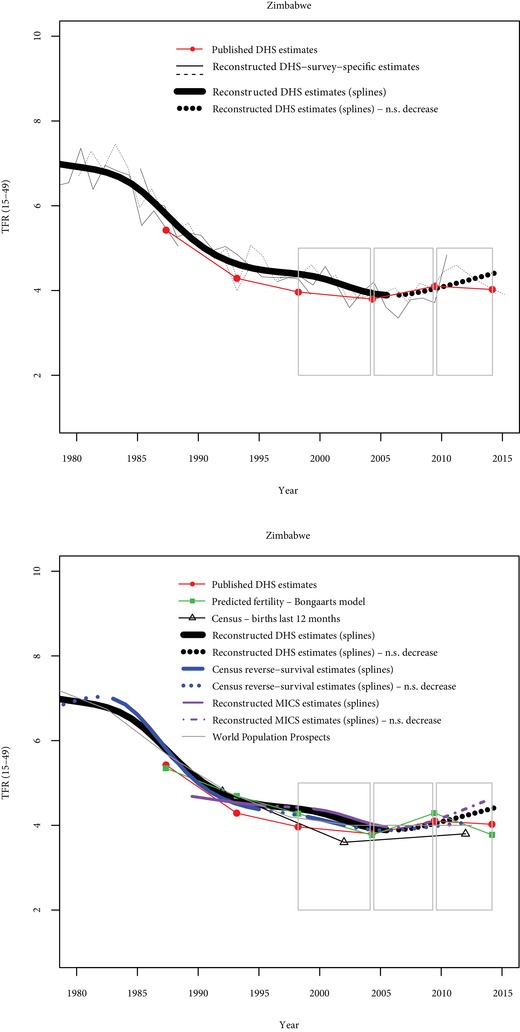
Comparisons of fertility trends across sources and methods in Zimbabwe N.S. decrease = Nonsignificant decrease (p>0.10). Grey rectangles indicate stalls identified from published DHS estimates.

**Figure 3:**
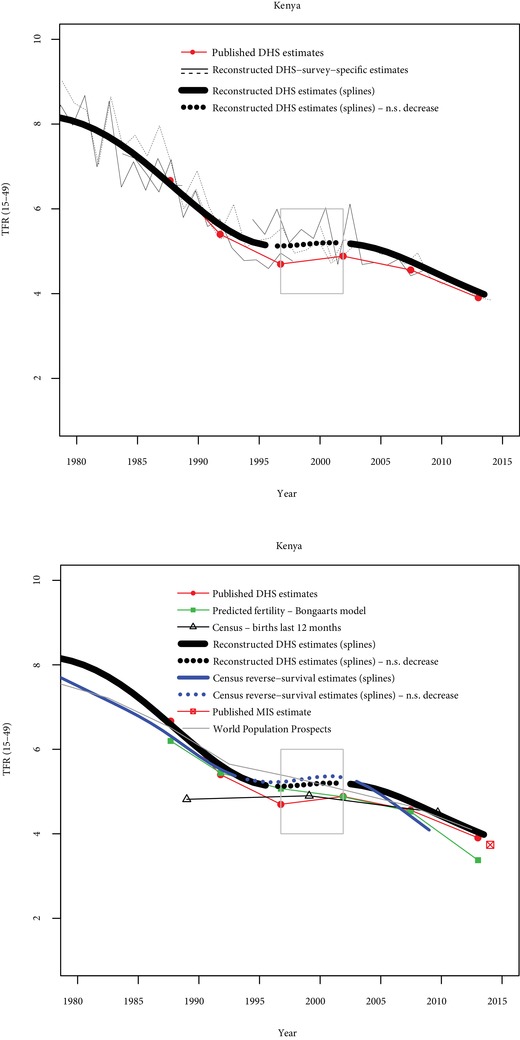
Comparisons of fertility trends across sources and methods in Kenya N.S. decrease = Nonsignificant decrease (p>0.10). Grey rectangles indicate stalls identified from published DHS estimates.

**Figure 4:**
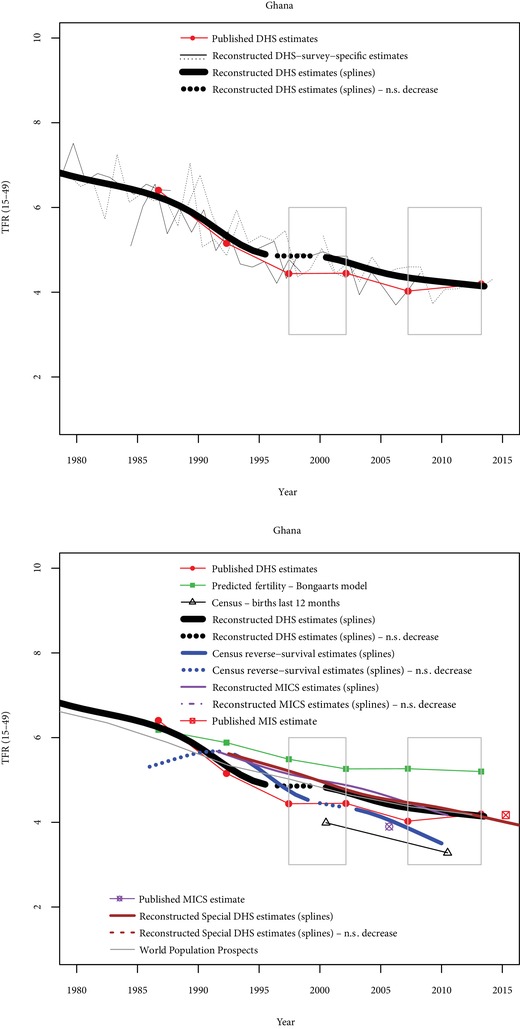
Comparisons of fertility trends across sources and methods in Ghana N.S. decrease = Nonsignificant decrease (p>0.10). Grey rectangles indicate stalls identified from published DHS estimates.

**Figure 5:**
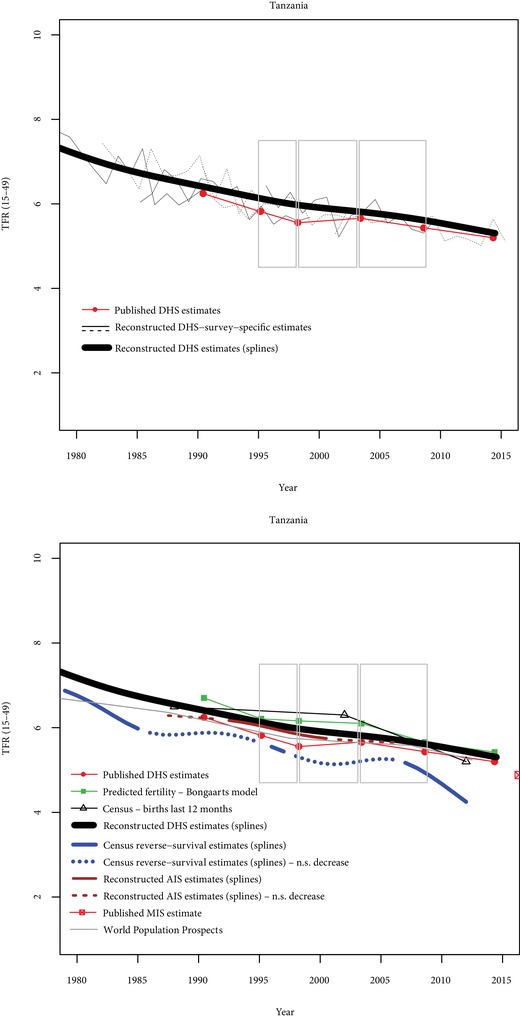
Comparisons of fertility trends across sources and methods in Tanzania N.S. decrease = Nonsignificant decrease (p>0.10). Grey rectangles indicate stalls identified from published DHS estimates.

**Figure 6:**
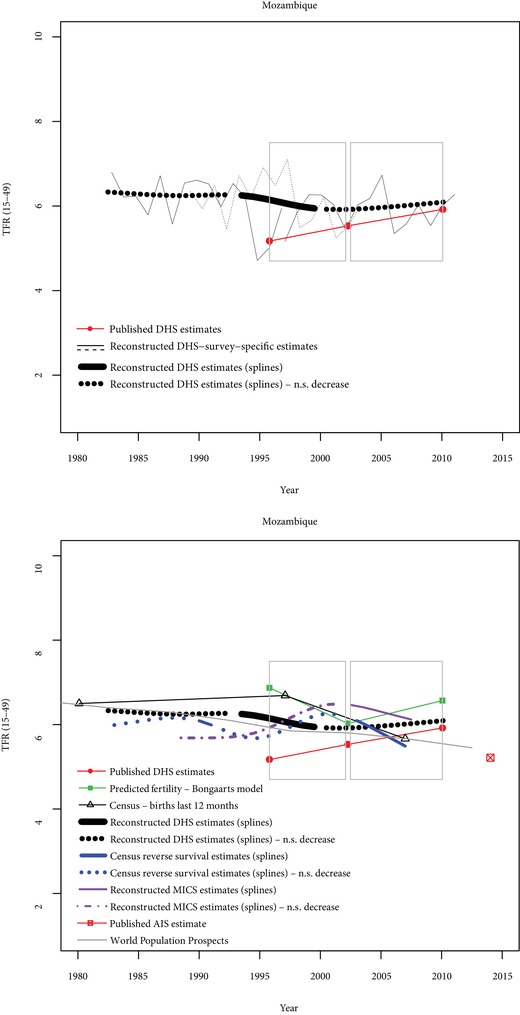
Comparisons of fertility trends across sources and methods in Mozambique N.S. decrease = Nonsignificant decrease (p>0.10). Grey rectangles indicate stalls identified from published DHS estimates.

Fertility trends are estimated in different ways, and consistency across the sources and methods is assessed through Figures 2 to 6, Appendix A,^2^ and the simple indices below. Fertility trends are first established using the total fertility rates (15–49) measured over the last three years, as published in DHS reports and on STATcompiler.^3^ These are used to identify a list of fertility stalls in the same way it is usually done (Bongaarts 2008; Shapiro and Gebreselassie 2008). These stalls are described in the next section.

Next, I reconstruct fertility trends from DHS birth histories by pooling two or more surveys. With good‐quality data, reconstructed trends from DHS birth histories and trends obtained with published TFRs should match (Schoumaker 2014). In contrast, data‐quality issues such as displacements of births, omissions of births, or differences in sample implementation may lead to inconsistencies across methods. Reconstructing trends from birth histories relies on creating tables of births and exposure by five‐year age groups and by single calendar year using the birth history data in each survey. Tables from several surveys are appended, and age‐specific fertility rates are estimated with Poisson regression, with age and time periods included as independent variables (Schoumaker 2013a).^4^ Restricted cubic splines are used to smooth fertility trends and identify stalls (Schoumaker 2013b, 2014).^5^ I use the same method with full birth histories available in MICS surveys.^6^ In most countries, only one MICS is available, and surveys are thus not pooled together; in two countries (Malawi and Zimbabwe), two surveys are pooled together. In Malaria Indicator Surveys (MIS), DHS interim or special surveys, and AIDS Indicator Surveys (AIS), TFRs are usually computed for the three years preceding the survey. In a few of these surveys, full birth histories are available, and fertility is reconstructed for the last 15 years using the same method as with the standard DHS.

I also use age–sex structures from census data to reconstruct fertility trends using the reverse‐survival method (Timæus and Moultrie 2013; Spoorenberg 2014). This method relies on the age distribution of children (aged 0–14) by single years of age, and on the numbers of women by five‐year age groups (among women aged 15–64). Both children and women are reverse‐survived to estimate the number of births and exposure by calendar year. I use the spreadsheet provided by Timæus and Moultrie (2013) to apply the reverse‐survival method, using the survival probabilities from the United Nations available in the spreadsheet (from 2015 World Population Prospects [WPP]). Yearly fertility estimates for the 15 years preceding each census are obtained, and restricted cubic splines are used to smooth fertility trends and to identify periods with no significant decline.^7^ The reverse‐survival method hinges strongly on the quality of the enumeration of children by age, which can be an issue in developing countries. The use of splines reduces the impact on fertility trends of heaping on specific ages, but data quality may lead to erratic trends in some cases. While I do not expect these estimates to match other estimates perfectly, high consistency with other sources would support the existence or absence of stalls.

Total fertility rates based on births in the last 12 months available in census data are also used. These estimates are probably less reliable than those based on other sources, as the number of births in the last 12 months is often underreported (Moultrie 2013). The way they may have been adjusted by the analysts is also not always clear from the census reports. Again, I do not expect these estimates to match estimates from other sources, but they may provide further evidence for stalls identified with other data sources and methods.

Two other sets of fertility estimates are presented in Figures 2 to 6. Contrary to the estimates discussed earlier, they are not used to identify the fertility stalls, but I will refer to these estimates in the discussion of the results and in the conclusion. First, I use the revised version of the Bongaarts model (Bongaarts 2015) to compute expected fertility levels and trends from the proximate determinants of fertility measured in Demographic and Health Surveys (sexual exposure, postpartum insusceptibility, contraceptive use). Three indices measure the fertility‐reducing effect of these proximate determinants, and the expected fertility level is estimated by multiplying the product of these three indices by 15.4 (the average total fecundity rate, Bongaarts 2015). Consistency between expected fertility from the proximate determinants and observed fertility should not be perfect, since expected fertility may be affected by measurement errors in the proximate determinants and by changes in unobserved proximate determinants, especially abortion. However, trends in expected fertility should be fairly consistent with trends in observed fertility with good‐quality data. I will refer to these estimates after identifying stalls with other sources. Secondly, I also present estimates from the United Nations WPP (United Nations Population Division 2017b) in the Figures 2 to 6, as these data have also been used to identify fertility stalls (Goujon, Lutz, and KC 2015). These estimates are obtained by combining a variety of sources (Gerland, Biddlecom, and Kantorová 2017) and are less erratic than estimates from a single source. They are not used to identify stalls in this article, but the stalls identified in this article will be compared to the WPP estimates.

## DEFINING AND IDENTIFYING STALLS

Stalls are first identified using the TFRs published in DHS reports. As discussed by Bongaarts (2008, p. 109), “a stall implies that an ongoing fertility transition is interrupted by a period of no significant change in fertility before the country reaches the end of the transition.” Two steps are thus necessary to identify countries where fertility has been stalling. First, a criterion must be used in considering that a fertility transition is underway. Second, one needs to measure the interruption of the decline in fertility.

In this article, I consider that fertility transition is underway if the published TFR (for the last three years) is at least 10 percent lower than the maximum average number of children ever born among women aged 45–49 in any preceding DHS survey, and if contraceptive prevalence among married women is at least 10 percent.^8^ By that definition, all but four African countries (DR Congo, Guinea, Mali, and Niger) have been in transition since the 1990s.^9^ To further refine the classification, I also distinguish early transitions from mid‐transitions; mid‐transition is reached when fertility has decreased below five children per woman (National Research Council 2000; Bongaarts 2006; Shapiro and Gebreselassie 2008). In this article, 13 countries are in the mid‐transition group at some point.

I distinguish three types of fertility changes. The first type corresponds to stagnating or increasing fertility between two successive surveys, i.e., countries where the published TFR is greater than or equal to the TFR in the previous survey. This approach is intuitive and simple to implement and is a conservative way of identifying fertility stalls. The second type of fertility change corresponds to fertility decreases that are not statistically significant (Bongaarts 2008).^10^ The third type of change includes situations of fertility decreases that are statistically significant.

Combining these two criteria leads to seven categories (Table 2): one category for pre‐transitional situations (no transition), three categories for early‐transition countries (no stall, slight stall, stall), and the same three categories for mid‐transition countries (Figure 1). The nine cases of stalls among mid‐transition countries include stalls in the late 1990s in Cameroon, Ghana, and Kenya, which were identified in the early analyses of fertility stalls in sub‐Saharan Africa (Westoff and Cross 2006; Bongaarts 2008; Shapiro and Gebreselassie 2008), as well as more recent stalls in Congo, Ghana, Namibia, Senegal, and Zimbabwe.^11^ Using a less conservative definition of stalls in mid‐transition countries brings in five additional cases of *slight stalls* (three additional countries: Ethiopia, Gabon, Lesotho), which have been discussed less often in the literature. Taking into account early‐transition countries with either fertility increases or nonsignificant decreases, the number of stalls reaches 37 cases in 23 countries (Table 3), that is, 40 percent of the 93 cases examined, in more than two‐thirds of the countries. In summary, the number of stalls clearly depends on the way transitions and fertility changes are measured.

**Table 2:** Classification of fertility changes, 32 countries, 93 cases (pairs of surveys)

	No stall (significant decline or nodecline in case of no transition)	Slight stall (no significantdecline)	Stall (stagnation or increasein fertility)
	56 cases, 25 countries	18 cases, 13 countries	19 cases, 16 countries
		37 cases, 23 countries	
	**7 cases, 4 countries**		
No transition	DR Congo (2007–2013), Guinea (1999–2005, 2005–2012), Mali (1987–1996, 1996–2001, 2001–2006), Niger (1992–1998)		
		**23 cases, 14 countries**	
	**39 cases, 20 countries**	**13 cases, 10 countries**	**10 cases, 9 countries**
Early Transition	Benin (1996–2001, 2006–2012), Burkina Faso (1999–2003), Burundi (1987–2010, 2010–2017), Cameroon (1991–1998), Ethiopia (2005–2010), Ghana (1988–1993, 1993–1998), Kenya (1989–1993, 1993–1998), Liberia (1986–2007, 2007–2013), Madagascar (1997–2004, 2004–2009), Malawi (1992–2000, 2000–2004, 2004–2010, 2010–2015), Mali (2006–2013), Namibia (1992–2000), Nigeria (1990–2003, 2008–2013), Rwanda (1992–2000, 2008–2011), Senegal (1986–1993, 1993–1997, 1997–2005, 2005–2011), Tanzania (1992–1996, 2010–2015), Togo (1988–1998, 1998–2014), Uganda (1988–1995, 2006–2011, 2011–2016), Zambia (1992–1996, 2007–2013), Zimbabwe (1988–1994).	Burkina Faso (1993–1999), Chad (1997–2004), Cote d'Ivoire (1994–1999, 1999–2012), Ethiopia (2000–2005), Madagascar (1992–1997), Niger (1998–2006), Sierra Leone (2008–2013), Tanzania (1996–1999, 2004–2010), Uganda (1995–2001, 2001–2006), Zambia (1996–2002).	Benin (2001–2006), Burkina Faso (2003–2010), Chad (2004–2015), Mozambique (1997–2003, 2003–2011), Niger (2006–2012), Nigeria (2003–2008), Rwanda (2000–2005), Tanzania (1999–2004), Zambia (2002–2007)
		**14 cases, 10 countries**	
	**10 cases, 9 countries**	**5 cases, 4 countries**	**9 cases, 7 countries**
Mid Transition	Comoros (1996–2012), Ghana (2003–2008), Kenya (2003–2009, 2009–2014), Lesotho (2004–2009), Namibia (2000–2007), Rwanda (2011–2015), Senegal (2013–2015), South Africa (1998–2016), Zimbabwe (1994–1999)	Ethiopia (2011–2016), Gabon (2000–2012), Lesotho (2009–2014), Zimbabwe (1999–2005, 2011–2015)	Cameroon (1998–2004, 2004–2011), Congo (2005–2011), Ghana (1998–2003, 2008–2014), Kenya (1998–2003), Namibia (2007–2013), Senegal (2011–2013), Zimbabwe (2005–2011).

**Table 3:** Number of cases of stalls and countries experiencing stalls using various definitions of stalls

Types of stalls	Cumulated number of countries	Cumulated number of cases
Mid‐transition stalls	7	9
+ Mid‐transition slight stalls	10	14
+ Early‐transition stalls	19	24
+ Early‐transition slight stalls	23	37

One should note that the stalls identified in this way are influenced by the duration between successive Demographic and Health Surveys. While in many cases DHS are spaced by around five years, much longer periods are sometimes found between successive surveys.^12^ Short stalls may thus not be identified with published data when the duration between two surveys is long.

## CONSISTENCY OF STALLS ACROSS DATA SOURCES AND METHODS

Depending on the criteria used to identify stalls and whether we focus on mid‐transition countries or also include early‐transition countries, the number of cases varies from 9 stalls in 7 countries to 37 stalls in 23 countries (out of 93 cases in 32 countries, Table 3). Some of these stalls may also reflect data‐quality issues. The quality of fertility data varies greatly from one place to another, and may also vary from one survey to another in the same country (Blacker 1994; Schoumaker 2014; Gerland, Biddlecom, and Kantorová 2017). As a result, some of these stalls may result from differential data quality across surveys (Schoumaker 2009; Machiyama 2010).

I combine two types of approaches to evaluate the genuineness of these stalls. First, internal consistency of DHS data is assessed by comparing fertility trends obtained from published estimates and fertility trends reconstructed from pooled DHS birth histories. The rationale for this is that with good‐quality data, these trends should be consistent (Schoumaker 2014). In contrast, inconsistencies reflect data‐quality issues. In summary, stalls identified with both published fertility and reconstructed trends are likely to be genuine. Second, external consistency is assessed by comparing DHS fertility trends with trends from the other sources (MICS, censuses, and other surveys). While these other sources may also be affected by data‐quality issues, stalls identified from these independent sources in addition to DHS data are likely to be genuine.

I combine the visual inspection of fertility trends with a more systematic coding of consistency across sources. A simple approach is used to summarize the information (see Appendix). A score varying from –1 to 1 is attributed to each of the 93 pairs of surveys for each source of information. The score is equal to 1 if the source indicates strong support for a stall, that is, a situation where fertility is stagnating or increasing over most of the period. Moderate support for fertility stall (score equal to 0.5) corresponds to a situation of nonsignificant decline or a situation of stagnation or increase during less than half of the period. The score is equal to –1 if the source indicates a sustained fertility decrease over the entire period, while a value of –0.5 indicates moderate support for the decrease (fertility goes down for part of the period). The score is equal to 0 in indeterminate situations, mainly when the data covers only a small part of the period.

I use these scores to compute two summary indices that vary between –1 and +1 for each of the 93 pairs of surveys. The first index measures internal consistency of DHS‐based estimates (D‐index, for DHS). It is the average score for the published DHS estimates and the reconstructed fertility trends from DHS. The index is equal to 1 if both published and reconstructed fertility trends from DHS show a stall, and it is equal to –1 if neither indicates a stall. The second index (O‐index, for other data sources), is equal to the average score for the estimates from other data sources (MICS, censuses, nonstandard DHS). It is equal to 1 if all the other sources consistently indicate a stall, and is equal to –1 if none of these other sources indicate a stall. These two indices are compared to assess the evidence regarding fertility stalls. In brief, stalls are considered to be strongly supported by the data if both the D‐index and the O‐index are close to 1, indicating high internal and external consistency.

This approach is illustrated with data from Zimbabwe, where six DHS are available, as well as two MICS and estimates from census data (Figure 2 and Table 4). The first panel of Figure 2 compares published DHS fertility estimates and reconstructed fertility estimates. Reconstructed yearly estimates for the 15 years preceding each survey separately are also displayed in this figure, as they provide useful information for understanding the differences between published estimates and reconstructed estimates. Nonsignificant declines (p>0.10) identified with restricted cubic splines are represented by dotted lines. On the second panel of Figure 2, estimates from other data sources are added, including from the Bongaarts model and WPP estimates. Table 4 shows the scores for each of the five pairs of surveys and each source of information.

**Table 4:** Consistency in fertility trends across sources in Zimbabwe

	**Period**				
	**1988–1994**	**1994–1999**	**1999–2005**	**2005–2011**	**2011–2015**
**DHS**					
Published DHS estimates	−1.0	−1.0	0.5	1.0	1.0
Reconstructed DHS estimates	−1.0	−1.0	−1.0	1.0	1.0
**D‐index (Consistency of DHS‐based estimates)**	**−1.00**	**−1.00**	**−0.25**	**1.00**	**1.00**
**Other data sources**					
Censuses (reverse survival)	−1	0	−0.5	1	0.5
Census (births last 12 months)	*No data*	−1	−0.5	1	0.5
Reconstructed MICS estimates	−0.5	0.5	−1	1	1
**O‐index (Consistency of other data sources)**	**−0.75**	**−0.17**	**−0.67**	**1.00**	**0.67**

NOTE: A value of 1 corresponds to strong support for a stall, and a value of –1 strong support for the absence of a stall.

In the late 1980s and early 1990s, published DHS estimates (red dots) indicate a significant decline (Figure 2). Three stalls (indicated by grey rectangles) are found with published DHS, from the late 1990s until the latest DHS in 2015. Reconstructed fertility trends are fairly consistent with published fertility trends, indicating good data quality. According to the reconstructed trends, fertility has stalled since 2006. The two most recent periods of stalling fertility identified with published data are thus confirmed with reconstructed data. The D‐index summarizes these results. It is equal to −1 in the first two periods (clear decline), +1 in the two most recent periods (clear stall), and −0.25 between 1999 and 2005. Three other sources are available in Zimbabwe to measure fertility trends (Figure 2): reconstructed trends from two MICS, reverse‐survival estimates from census data, and census estimates based on births in the last 12 months. In the first period (between the 1988 and 1994 DHS), two sources are available and indicate a fertility decline (O‐index=−0.75). Between 1994–1999 and 1999–2005, the three sources are not perfectly consistent, but suggest that fertility decreased (O‐index=−0.17 between 1994 and 1999, and O‐index=−0.67 between 1990 and 2005). In contrast, in the two most recent periods, these other sources indicate that fertility stalled. Between 2005 and 2011, all three sources indicate a stall, and the O‐index is at its maximum (O‐index=1). Between 2011 and 2015, these sources suggest a stall, but their consistency is not perfect (O‐index=0.67). Fertility trends predicted from the Bongaarts model are consistent overall with trends from other sources, but not for the most recent period. In summary, these data provide unambiguous evidence for a stall in Zimbabwe between the 2005 and 2011 surveys (both the D‐index and the O‐index are equal to 1), and strong evidence for one between 2011 and 2015.

Four other cases are briefly discussed: Kenya, Ghana, Tanzania, and Mozambique. In Kenya, six DHS are also available (Figure 3), in addition to census data (reverse‐survival estimates and births in the last 12 months). The stall between the 1998 and 2003 surveys is strongly supported by the data (D‐index=1.0, O‐index=0.75). Six DHS surveys are also available in Ghana, and two stalls are found with published estimates, one in the late 1990s, and the second one between the two most recent surveys (Figure 4). The first stall is also supported using reconstructed trends from DHS data (D‐index=1), but not by other data sources (O‐index=−0.37), indicating conflicting evidence. The second stall is not found with reconstructed trends (D‐index=0), and not supported by other data sources (O‐index=−0.5), indicating weak evidence for the stall.

In Tanzania (Figure 5), three stalls are found with published data, and only one of them corresponds to an increase in the TFR. None of these stalls is found with reconstructed fertility trends from DHS (D‐indices equal to zero or −0.25). The stall between 1999 and 2004 is somewhat supported by reverse‐survival estimates from census data (O‐index=0.5), and the Bongaarts estimates are also consistent with a fertility increase; the 1996–1999 stall is not supported by non‐DHS data (D‐index=−0.5 and O‐index=−0.17) and evidence is also very weak for the 2004–2010 stall (D‐index=−0.5 and O‐index=−0.17).

Finally, the Mozambique case (Figure 6) illustrates a pretransitional situation rather than a situation of stalling fertility. Reconstructed fertility trends indicate that fertility has remained stable over a 25‐year period, and that published fertility estimates seem underestimated. Other sources (reverse‐survival estimates from censuses and MICS) also indicate that fertility is higher than published DHS estimates, and no previous significant decline is visible from these data.

In summary, these five countries illustrate that some stalls are strongly supported by a variety of data sources (e.g., Zimbabwe 2005–2011 and, to a lesser extent, Kenya 1998–2003). Some stalls seem to be spurious, either because no previous decline was found (e.g., Mozambique 1997–2003), or because the slowdown in fertility decline is not confirmed by other sources (e.g., Tanzania 2004–2010). Finally, some of the stalls are supported by some sources but not by others (e.g., Ghana 1998–2003).

The same exercise is performed for the 93 pairs of surveys (Appendix B). In 15 cases, fertility transition had not started. Evidence on fertility stalls is assessed for the remaining 78 pairs of surveys. For each of these pairs, I combine the D‐index and O‐index to classify the cases into six categories (Table 5).

**Table 5:** Classification of fertility stalls based on DHS data and other sources (78 cases)

		O‐index (other sources)				
		No other source	<0(no support)	Between 0 and 0.49(moderate support)	Between 0.50 and 0.99(strong support)	1(very strong support)
**D‐index (DHS)**	**<0** **(no support)**	6 cases	32 cases	8 cases	3 cases Kenya (1993–1998), Senegal (2005–2011), Uganda (2001–2006)	2 cases Ghana (1988–1993), Senegal (1993–1997)
	**Between 0 and 0.49** **(moderate support)**	0 cases	6 cases	6 cases Benin (2001–2006), Burkina Faso (1993–1999), Malawi (2000–2004), Rwanda (2000–2005), Cameroon (2004–2011), Zambia (1992–1996)	5 cases Tanzania (1999–2004), South Africa (1998–2016), Nigeria (1990–2003; 2003–2008), Côte d'Ivoire (1999–2012)	0 cases
	**Between 0.50 and 0.99** **(strong support)**	0 cases	0 cases	2 cases Gabon (2000–2012), Madagascar (1992–1997)	0 cases	0 cases
	**1** **(very strong support)**	0 cases	1 case Ghana (1998–2003)	1 case Cameroon (1998–2004)	4 cases Congo (2005–2011), Kenya (1998–2003), Zambia (2002–2007), Zimbabwe (2011–2015)	2 cases Zimbabwe (2005–2011), Namibia (2007–2013)

NOTE: The following 15 cases are not included, because—based on reconstructed fertility trends with DHS—fertility decline had not started by the beginning of the period: Chad (1997–2004, 2004–2015), DR Congo (2007–2013), Guinea (1999–2005, 2005–2012), Mali (1987–1996, 1996–2001, 2001–2006), Mozambique (1997–2003, 2003–2011), Niger (1992–1998, 1998–2006, 2006–2012), Uganda (1986–1995, 1995–2001).

### Unambiguous Evidence (Black Cell, 2 Cases)

These stalls are strongly supported by DHS data (both published and reconstructed trends), and by all the other independent data sources available (surveys or censuses). Two stalls fall in this category: Zimbabwe (2005–2011) and Namibia (2007–2013). The stalls in Zimbabwe and Namibia are also consistent with trends in proximate determinants, and are also found in the WPP.

### Very Strong Evidence (Dark Gray Cells, 4 Cases)

This corresponds to situations where DHS and other sources strongly support the stalls (Congo 2005–2011, Kenya 1998–2013, Zambia 2002–2007, and Zimbabwe 2011–2015), but not unambiguously (D‐index=1 and O‐index above 0.5, or O‐index=1 and D‐index above 0.5). In Congo and Zimbabwe, trends from the WPP are consistent with the stalls; in contrast, stalls are not visible in Kenya and Zambia in the WPP. Trends in proximate determinants are consistent with stalls in Congo and in Zambia.

### Strong Evidence (Medium Gray Cells, 1 case)

This category includes only one case, where DHS strongly support the stall and the other sources moderately support it (Cameroon 1998–2004). However, it is not found in the WPP, and the trend in the proximate determinants does not support it.

### Moderate Evidence (Light Gray Cells, 7 Cases)

These cases correspond to situations with strong support for stalls in at least one type of source (D‐index or O‐index between 0.5 and 0.99), and at least moderate support for the other source(s) (D‐index or O‐index>0). They include stalls in Côte d'Ivoire, Gabon, Madagascar, Nigeria, South Africa, and Tanzania. WPP estimates do not indicate stalls in these countries, although they suggest a slowdown in the case of South Africa. Trends in proximate determinants are clearly consistent with stalls in Côte d'Ivoire, Gabon, and South Africa.

### Weak or Conflicting Evidence (Very Light Gray Cells, 12 Cases)

In these situations, moderate support for the stalls is found in both types of sources, or no support is found in one type of source and there is at least strong support in the other source(s). This includes the stall in Ghana (1998–2003), as well as other stalls in a variety of countries. While there may be some stalls in these countries, they are not strongly supported by the data. They are also not supported by the WPP. Proximate determinants support stalls in some cases (e.g., Benin) but not in others (e.g., Ghana, Rwanda).

### Very Weak or No Evidence (White Cells, 52 Cases)

This is the largest category, containing 38 cases with no evidence for stalls and 14 cases with only mild evidence from one of the two types of sources. They represent two‐thirds of the 78 cases examined. No stalls are found in the WPP in these cases, and proximate determinants are usually consistent with decreasing fertility.

In summary, I find very strong support for stalls in five countries (Congo, Kenya, Namibia, Zambia, Zimbabwe). Fairly strong support is also found in Cameroon, in the early 2000s. Stalls are possible in seven cases from six other countries (Côte d'Ivoire, Gabon, Madagascar, Nigeria, South Africa, and Tanzania), where evidence is moderate. They cannot be ruled out in seven other countries (Benin, Burkina Faso, Ghana, Malawi, Rwanda, Senegal, and Uganda), but the evidence is limited or conflicting.

## DISCUSSION AND CONCLUSION

In this article, a variety of data sources and methods are used to identify fertility stalls. Using published TFRs from DHS reports and a broad definition of stalls (including early‐transition countries and slight fertility declines that are not significant), as many as 37 situations of stalling fertility in 24 countries are found; focusing on mid‐transition stalls brings their number down to 14 in 10 countries, and a more conservative definition (stagnation or increase in fertility in mid‐transition countries) leads to 9 stalls in 7 countries. Comparing fertility trends based on published DHS estimates to those using other methods and data sources leads to different conclusions and, not surprisingly, to a smaller number of cases. Unambiguous support is found for 2 stalls and very strong support for 6 stalls, and 7 stalls are at least fairly strongly supported by the data. Including stalls with moderate evidence, we reach a total of 14 stalls, and counting stalls with weak or conflicting evidence brings this number to 26.

The classification provided in this article is partly subjective and may certainly be improved, but it provides a more nuanced approach to identifying fertility stalls than the one based on simple comparisons of published TFRs. It also clearly leads to a smaller number of stalls than those identified with published DHS data. To be sure, five of the nine cases of mid‐transition stalls found by comparing TFRs from successive DHS (Table 2) are strongly supported by the various data sources. Some of these stalls, however, appear to be less trustworthy when various data sources are taken into consideration (e.g., Ghana 1998–2003 or Senegal 2011–2013). Stalls identified with published TFRs from DHS with a less conservative definition (nonsignificant declines, including in early‐transition countries) are often not strongly supported by the data. In short, their numbers tend to be inflated, because fertility either had not stopped decreasing or had not started decreasing in the first place. In contrast, the numbers of stalls found with the WPP is even smaller than those we find by comparing various data sources. While the Zimbabwe and Namibia stalls are also identified with the WPP, the Kenya stall (1998–2003), for instance, is not visible in the WPP. This may be due to the short duration of the stall and the fact that fertility trends are smoothed in the WPP, as they are averaged over five‐year periods. Overall, the WPP tend to be very conservative in identifying fertility stalls.

In the end, comparing various methods and sources helps identify cases that may be targeted for in‐depth research on the dynamics and causes of the stalls. Zimbabwe and Namibia are clear candidates for further research (Palamuleni 2015; Sayi 2015), as there is almost no doubt they have experienced fertility stalls. Congo and Zambia would also be interesting case studies, in addition to Kenya, which has already been fairly well documented (Westoff and Cross 2006; Magadi and Agwanda 2010). More limited evidence in other cases suggests that stalls either are slight and/or lasted only a short period of time, or that data‐quality issues may hamper further research on these cases.

Other stalls may be found with additional data or other methods. In some situations, the duration between successive surveys is very long, which may have masked clear stalls. This may be the case in South Africa, where reconstructed fertility trends from DHS and census data suggest a stall, while published TFRs from DHS indicate that fertility decreased slightly.^13^ This may also be the case in Côte d'Ivoire between 1999 and 2012. While these two countries are included in the moderate‐evidence category, stronger evidence could have been found with additional surveys. Other countries that were not included in this study—because at least two DHS were not available—may also have experienced stalls. For example, census data from Botswana suggest that fertility has stalled at around three children from the early 2000s, but no DHS or MICS are available to confirm this, and the WPP do not indicate such a stall.^14^ Finally, while this article focused on stalls at the national level, stalls may be found in urban areas without occurring in rural areas or at the national level. Conversely, stalls at the national level do not necessarily mean that stalls occur at subnational levels, as they may reflect composition effects (Sandron 2010; Eloundou‐Enyegue, Giroux, and Tenikue 2017). Exploring trends at the subnational level was beyond the scope of this article, but, as demonstrated by the analysis of stalls by levels of education (Kebede, Goujon, and Lutz 2019), it is a useful step in understanding the dynamics and the causes of fertility stalls.

In the end, despite the uncertainty surrounding some stalls, our results also indicate that they are not widespread but also not uncommon in sub‐Saharan Africa. They are also not a feature of the past, as shown by the ongoing stalls in Namibia and Zimbabwe. A better insight into their causes, such as the role of stalls in contraception and persisting high demand for children (Agyei‐Mensah 2005; Westoff and Cross 2006; Gillespie et al. 2007; Bongaarts 2008; Ezeh, Mberu, and Emina 2009), is thus key to understanding and influencing the future paths of fertility in sub‐Saharan Africa.

## Supporting information

Supporting information Appendix A

Supporting information Appendix B

## Footnotes

1 Reports are mainly available from statistical offices’ websites, census and demographic survey data archives (IREDA, ceped.org/ireda), and the International Household Survey Network (IHSN, http://catalog.ihsn.org). Data on age structure are also available from the United Nations Statistics Division (data.un.org/Data.aspx?d=POP&f=tableCode%3a22). Microdata from the Integrated Public Use Microdata Series (IPUMS, www.ipums.org) are used in a few cases where published data is not available (Minnesota Population Center 2015). TFRs from births in the last 12 months were also obtained from United Nations World Fertility Data (United Nations Population Division 2017a).

2 Appendixes are available at the supporting information tab at wileyonlinelibrary.com/journal/sfp.

3 Actually, TFRs and their standard errors are computed for the three years preceding the survey using the tfr2 Stata command (Schoumaker 2013a). These are identical to the TFRs published on STATcompiler and in DHS reports. Using the tfr2 command allows the computing of standard errors for all the surveys.

4 The age pattern of fertility is considered to be constant for each survey. A pattern of proportionate age‐specific fertility rates for each survey is computed; the pattern is multiplied by exposure and controlled for in the offset (Schoumaker 2014).

5 Restricted cubic splines are piecewise polynomial functions constrained to join at predefined years (knots) (Andersen 2009). Cubic splines are flexible and allow a large variety of shapes to be fitted with relatively few parameters (Harrell 2001). In this case, knots were spaced by five years.

6 I use data for the 20 years preceding the survey in the MICS, and for the 15 years preceding the survey for the DHS. Using a long period with MICS allows for comparisons with DHS over longer periods.

7 When several censuses are available, all estimates are used together for the smoothing of fertility trends. The method of smoothing (restricted cubic splines) is similar to the one used with birth histories, with knots spaced by five years.

8 This is based on the common threshold (10 percent decrease in fertility) to identify the onset of the fertility transition (Bongaarts 2008). Bongaarts (2008) considers the decline has started if contraceptive prevalence among married women is 10 percent or less, because of lack of data on past fertility. Gendell's approach (1985) is more conservative, as he limits his analyses to countries where fertility had decreased by at least 20 percent, and where the decline had been rapid (at least 0.15 children per year for at least 5 years), followed by a stop or a deceleration in the decline for at least 4 to 5 years.

9 The number of countries that have not started their transition will be revised upward later in this article.

10 Standard errors of TFRs are computed taking account of clustering using the jackknife method (Schoumaker 2013a). A one‐tailed *t*‐test is used to test the significance of fertility decrease (p<0.10).

11 For the stall in Zimbabwe, see Sayi (2015), Ndagurwa and Odimegwu (2019), Goujon, Lutz, and KC (2015), and Ezeh, Mberu, and Emina (2009); for the stall in Namibia, see Palamuleni (2015).

12 In two‐thirds of the cases, the duration between successive surveys is between five and seven years, and in 85 percent of the cases, it is between four and eight years. But in ten cases, the duration is greater than or equal to ten years, most notably in South Africa (between 1998 and 2016) and Burundi (between 1987 and 2010), but also Chad, Comoros, Côte d'Ivoire, Gabon, Liberia, Nigeria, and Togo.

13 The 2003 DHS was not used in this article as the data files were not available and the TFR was not published on STATcompiler. However, the TFR published in the report of the 2003 survey (Department of Health, Medical Research Council, ORC Macro 2007) suggests fertility stalled between the 2003 and 2016 surveys.

14 No clear signs of stalls were found in other countries, including in Eswatini (previously known as Swaziland), where two MICS suggest a sustained fertility decline from 1990 to 2014.
