# Supplementary material for: Stalls in Fertility Transitions in sub‐Saharan Africa: Revisiting the Evidence
Source: Stud Fam Plann. 2019 Aug 5;50(3):257–78. doi: 10.1111/sifp.12098 (PMC6771655; doi:10.1111/sifp.12098)
Supplement: Supplementary file 1 — Supporting information Appendix A [file SIFP-50-257-s001.docx]

**APPENDIX A**

Figure A1: Comparisons of fertility trends across data sources and methods in 32 sub-Saharan African countries

| 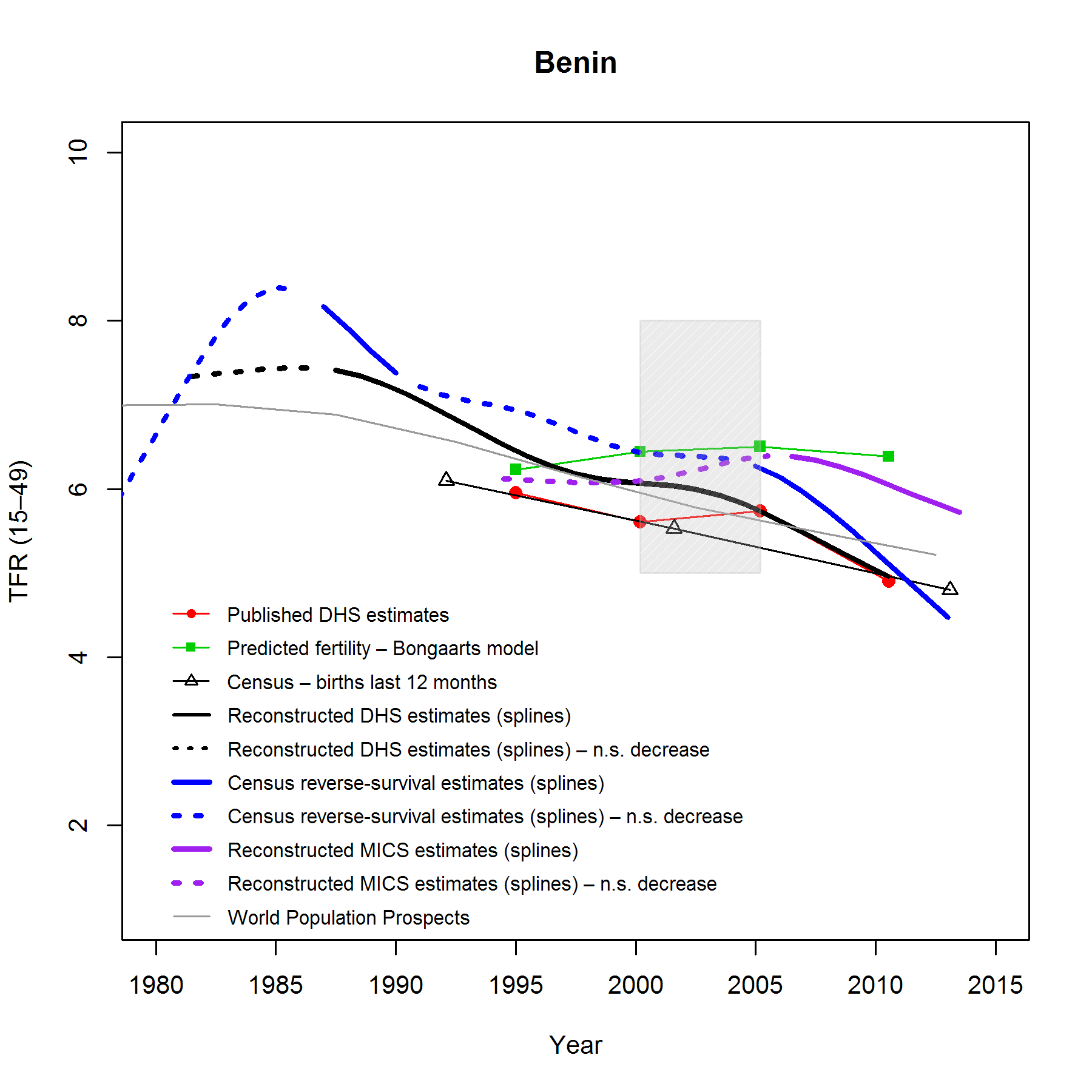 | 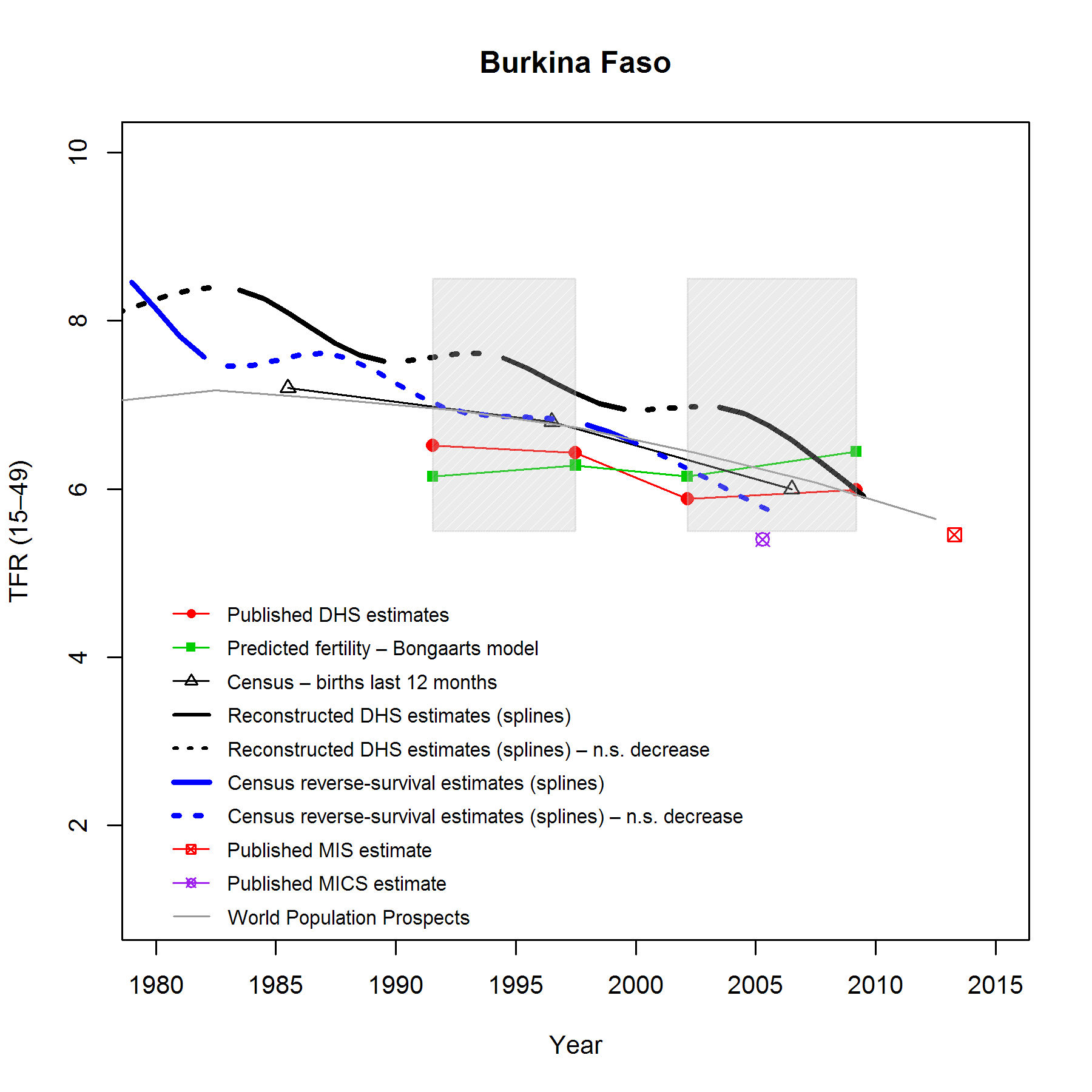 |
| --- | --- |
| 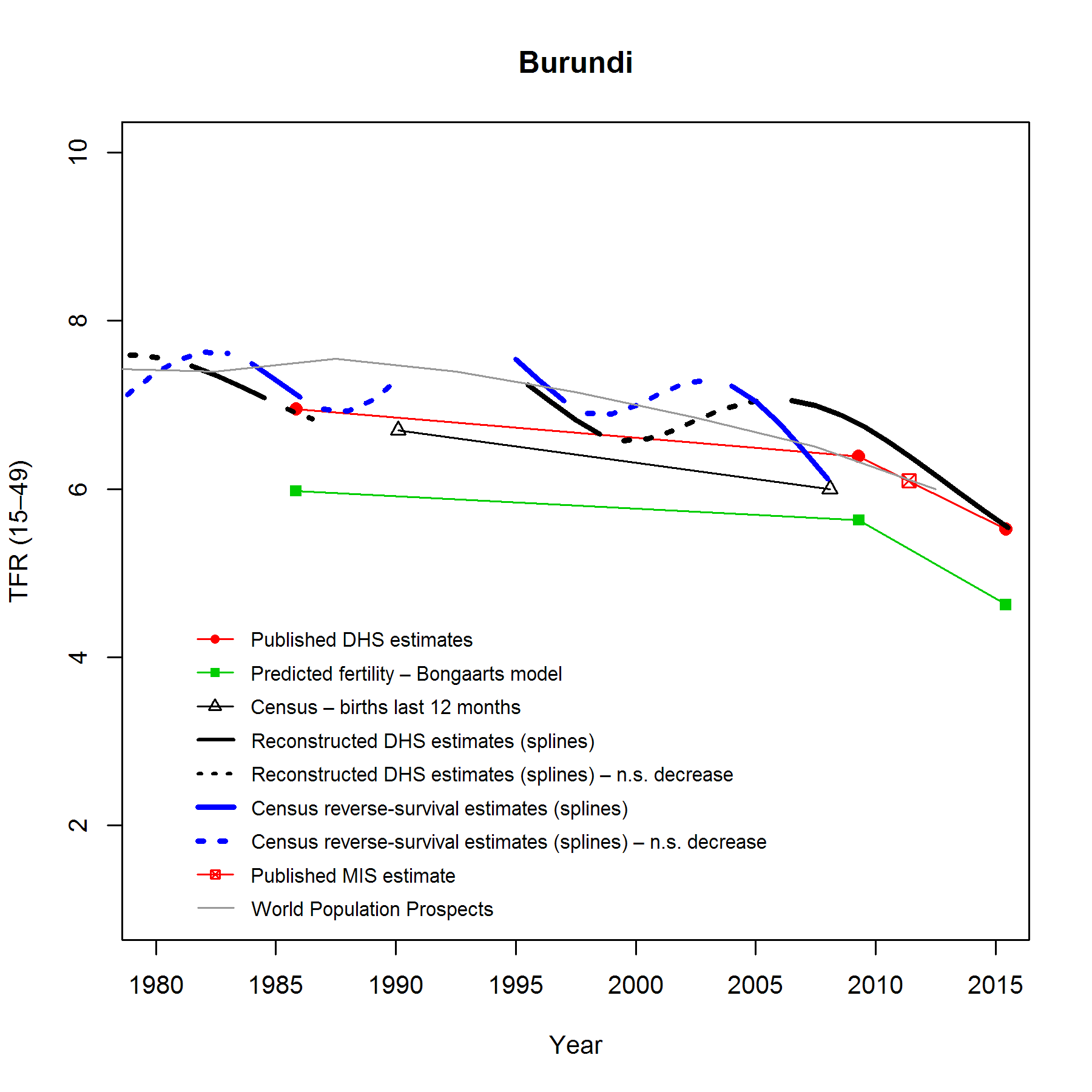 | 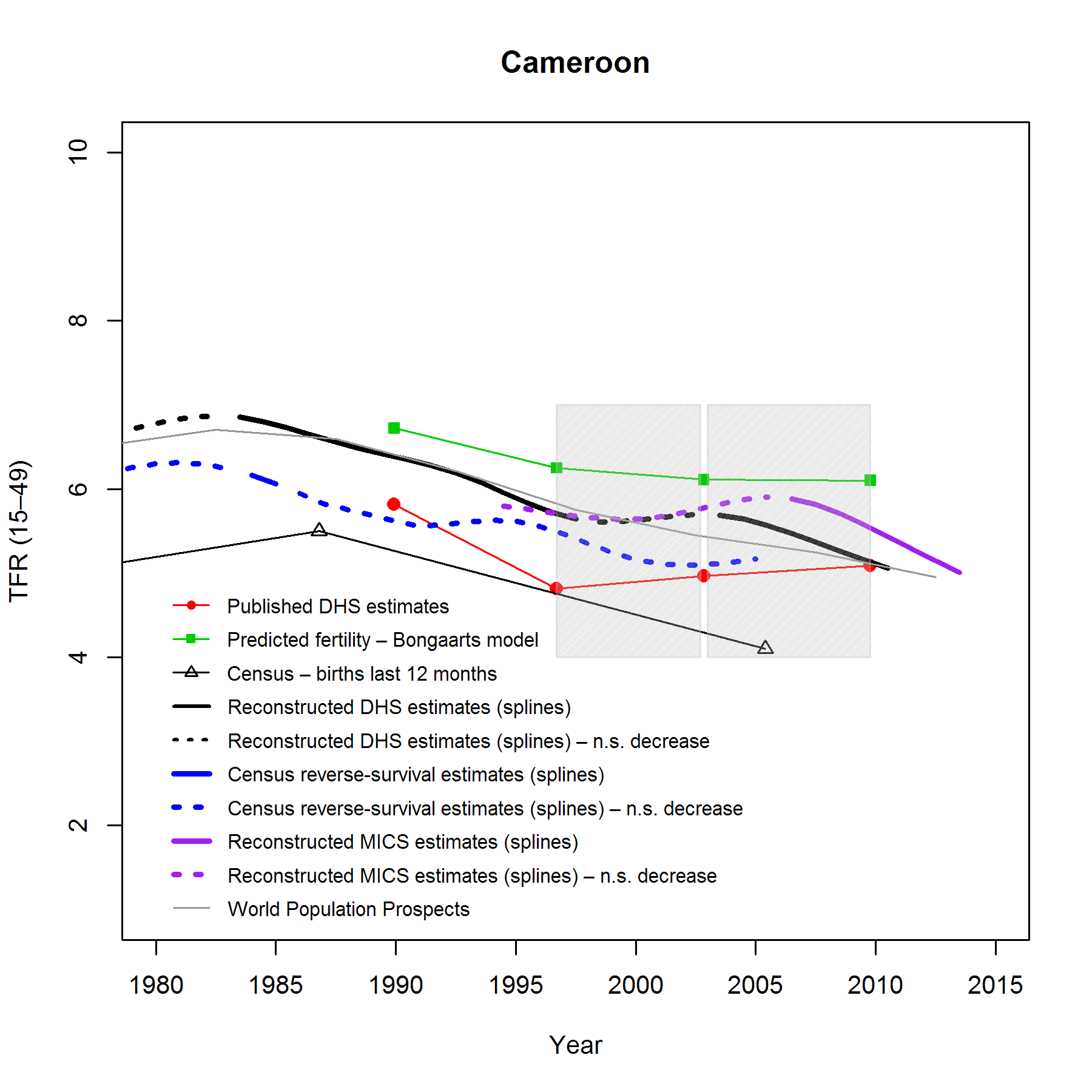 |
| 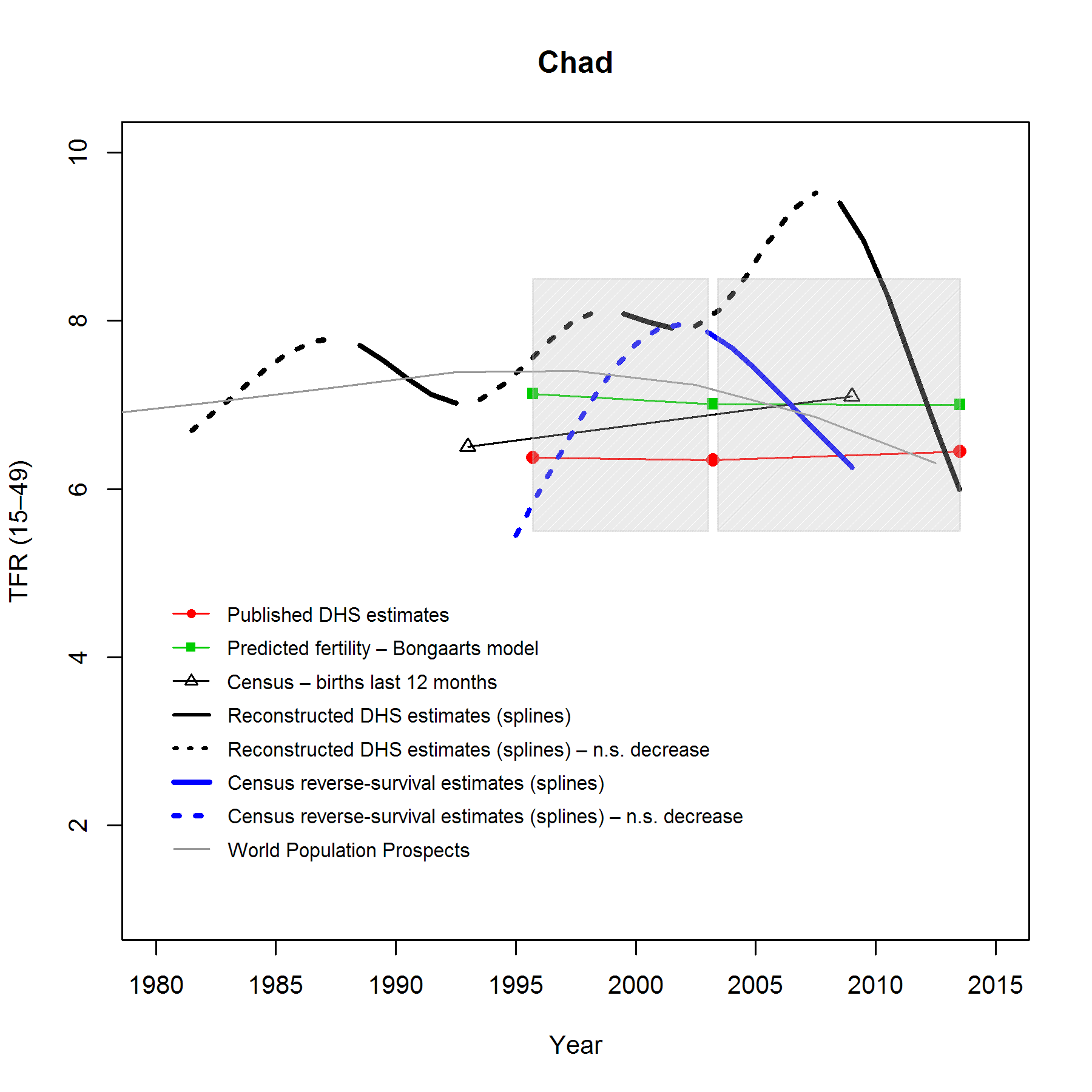 | 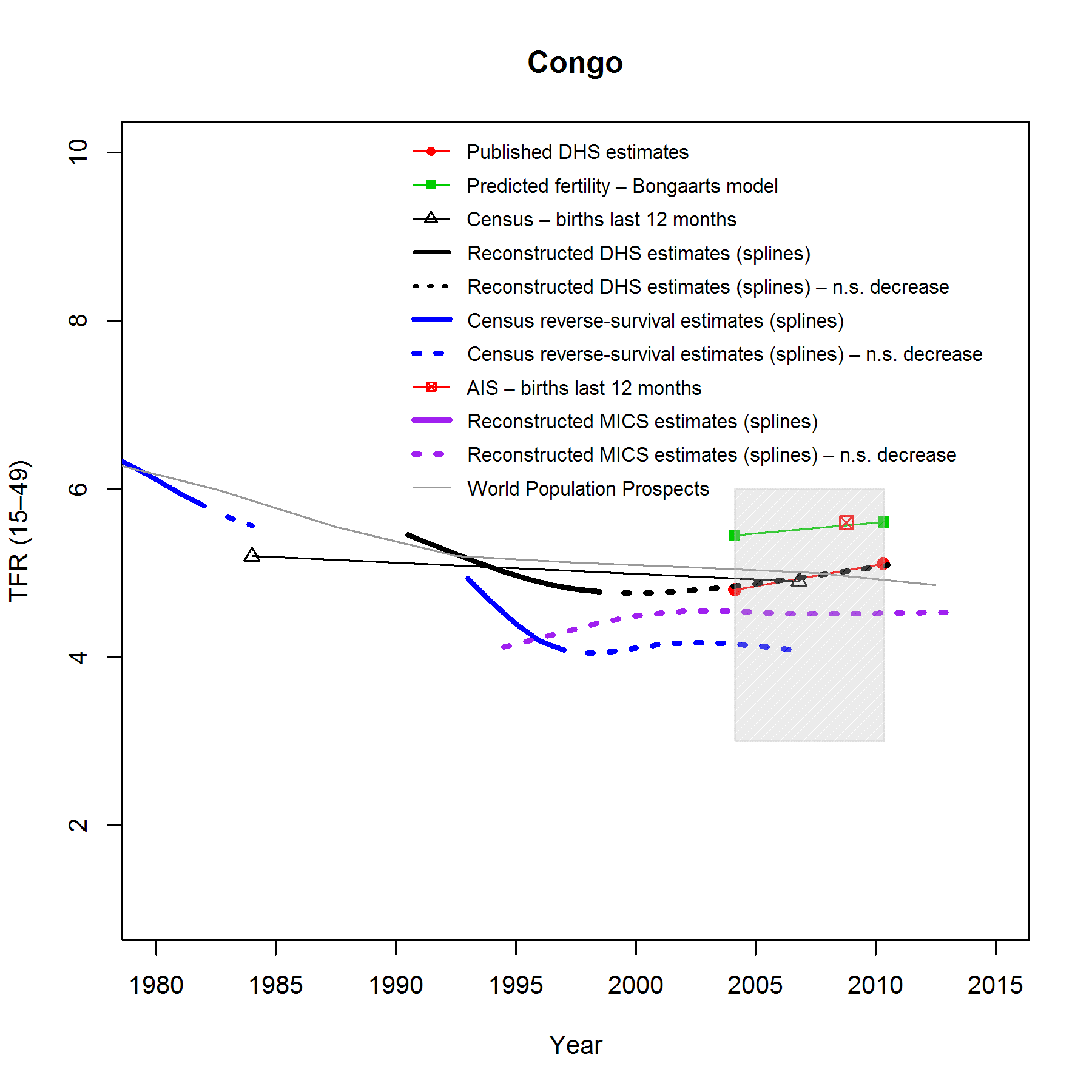 |
| 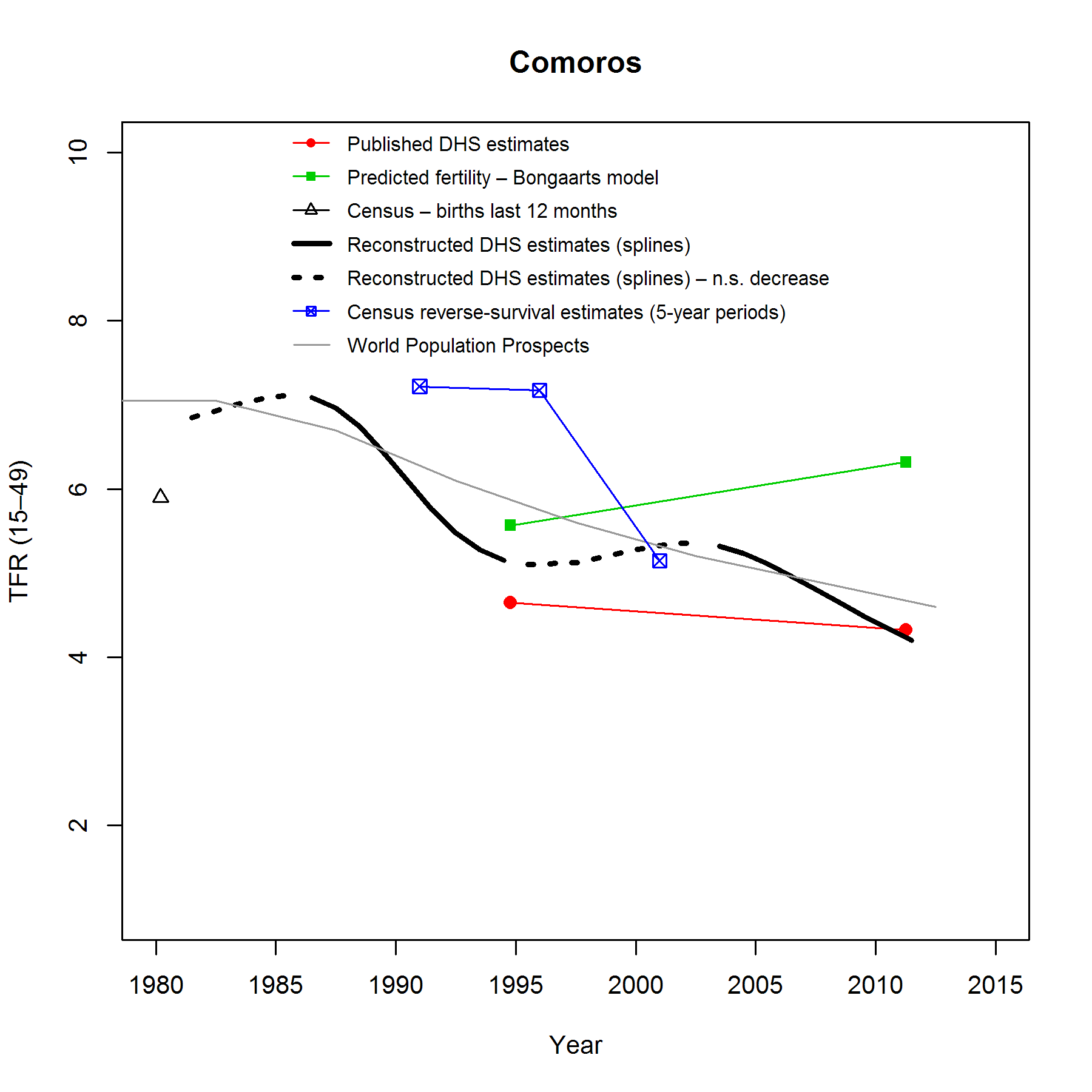 | 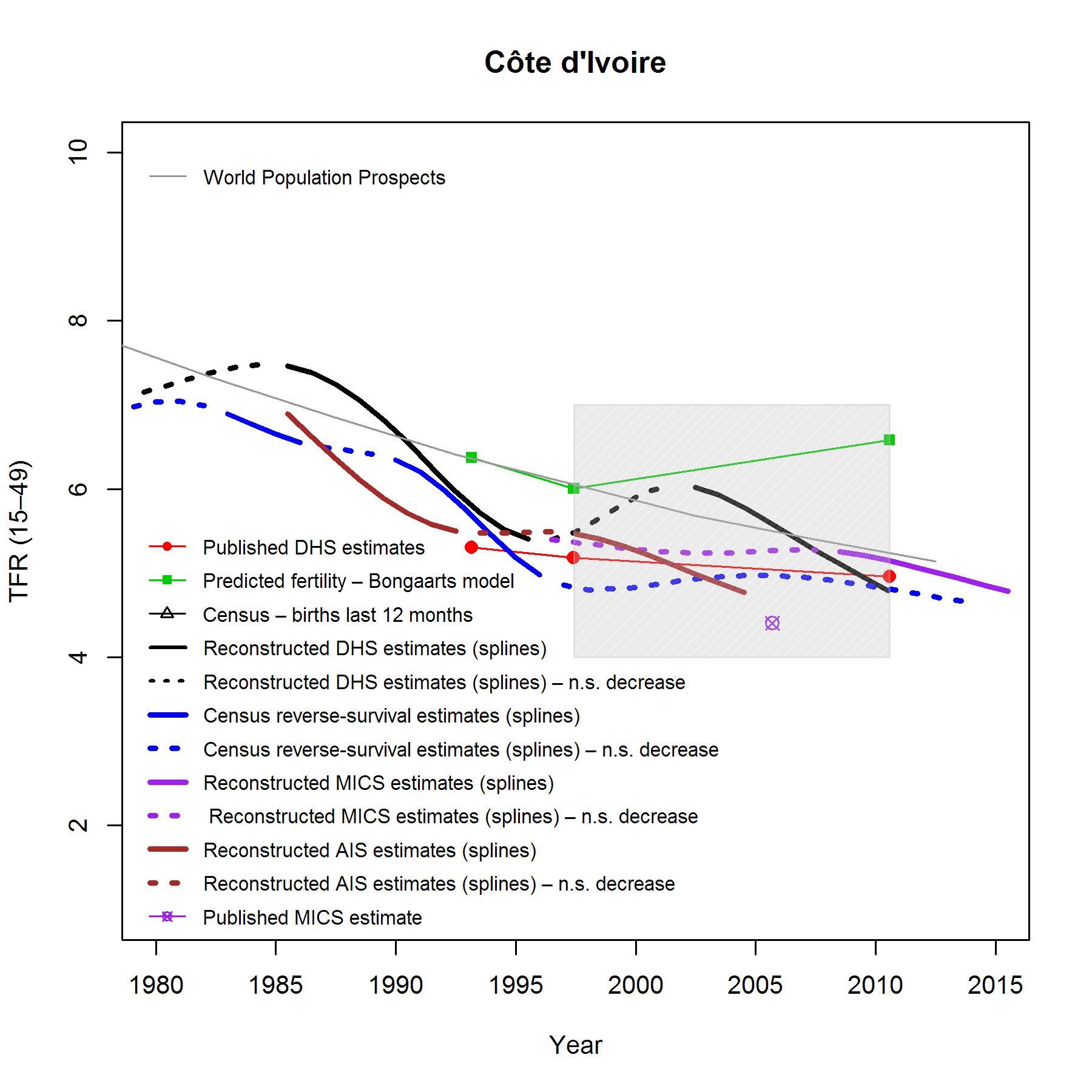 |
| 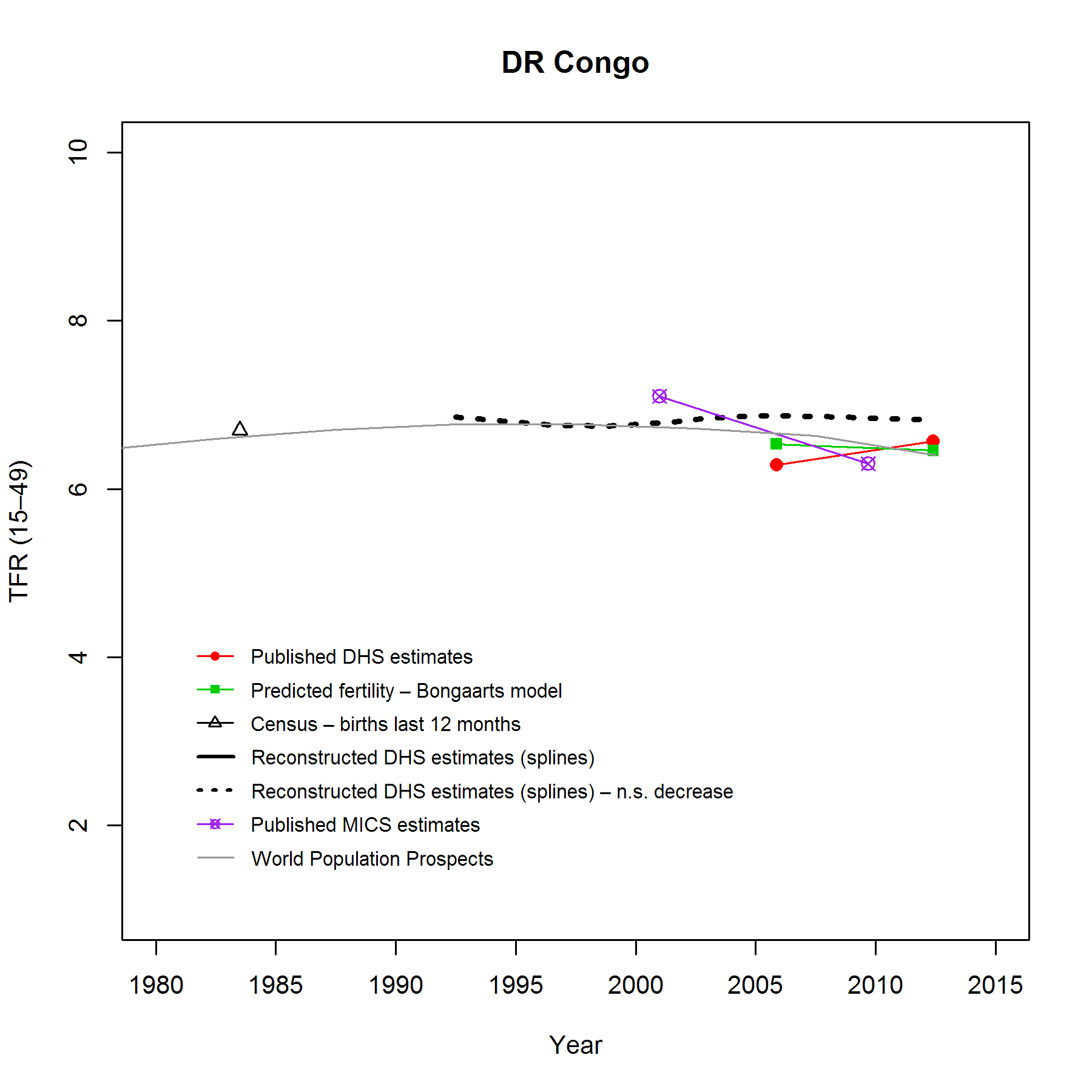 | 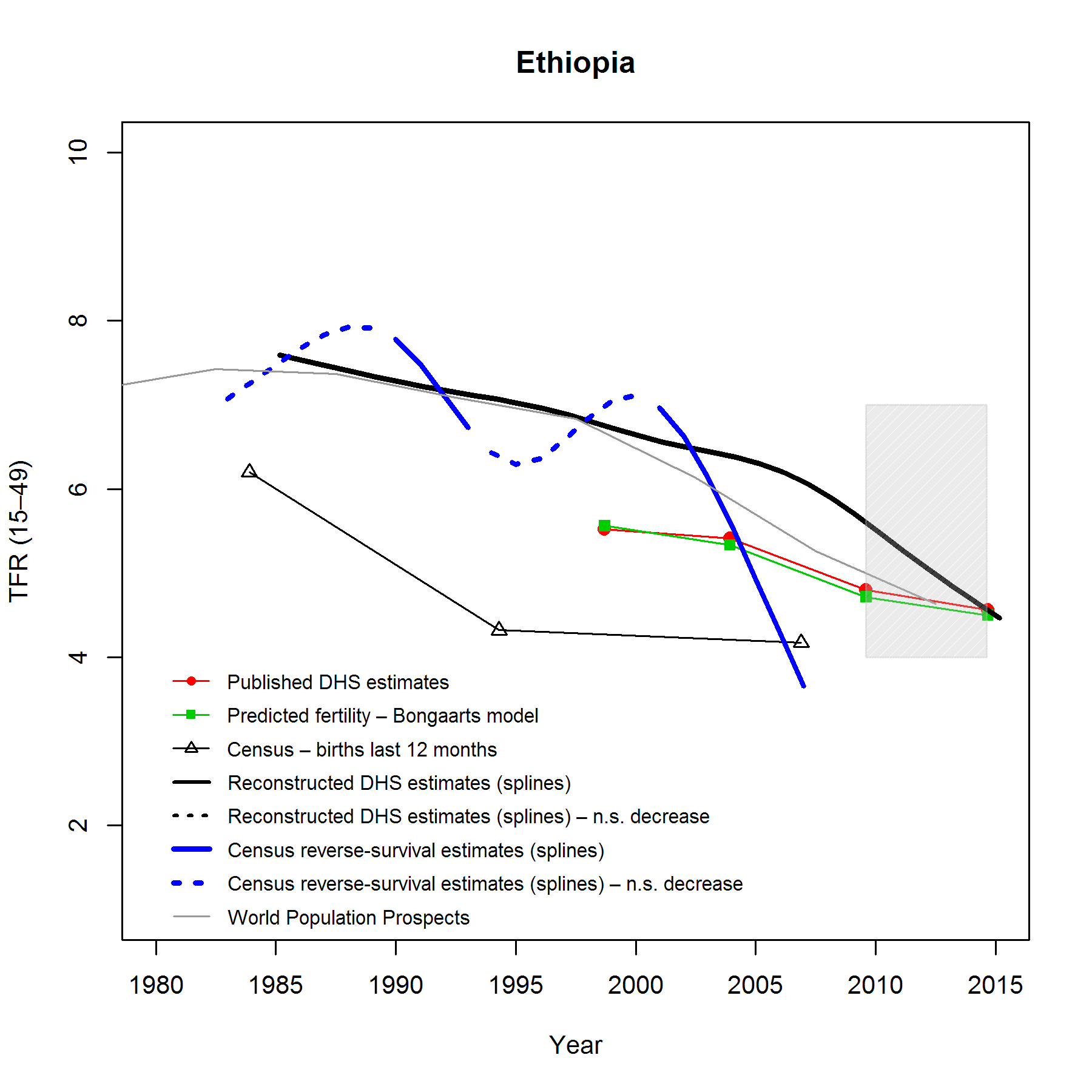 |
| 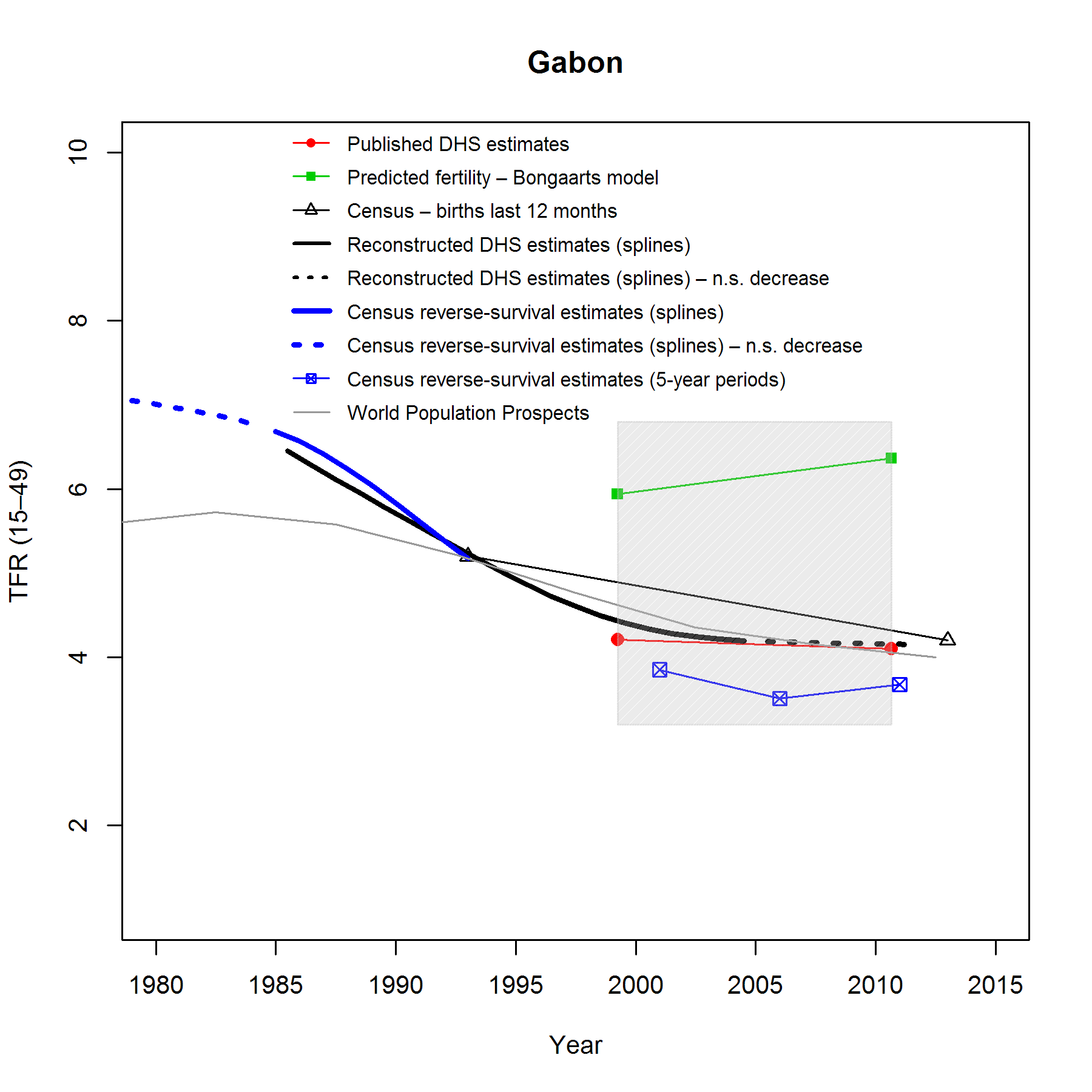 | 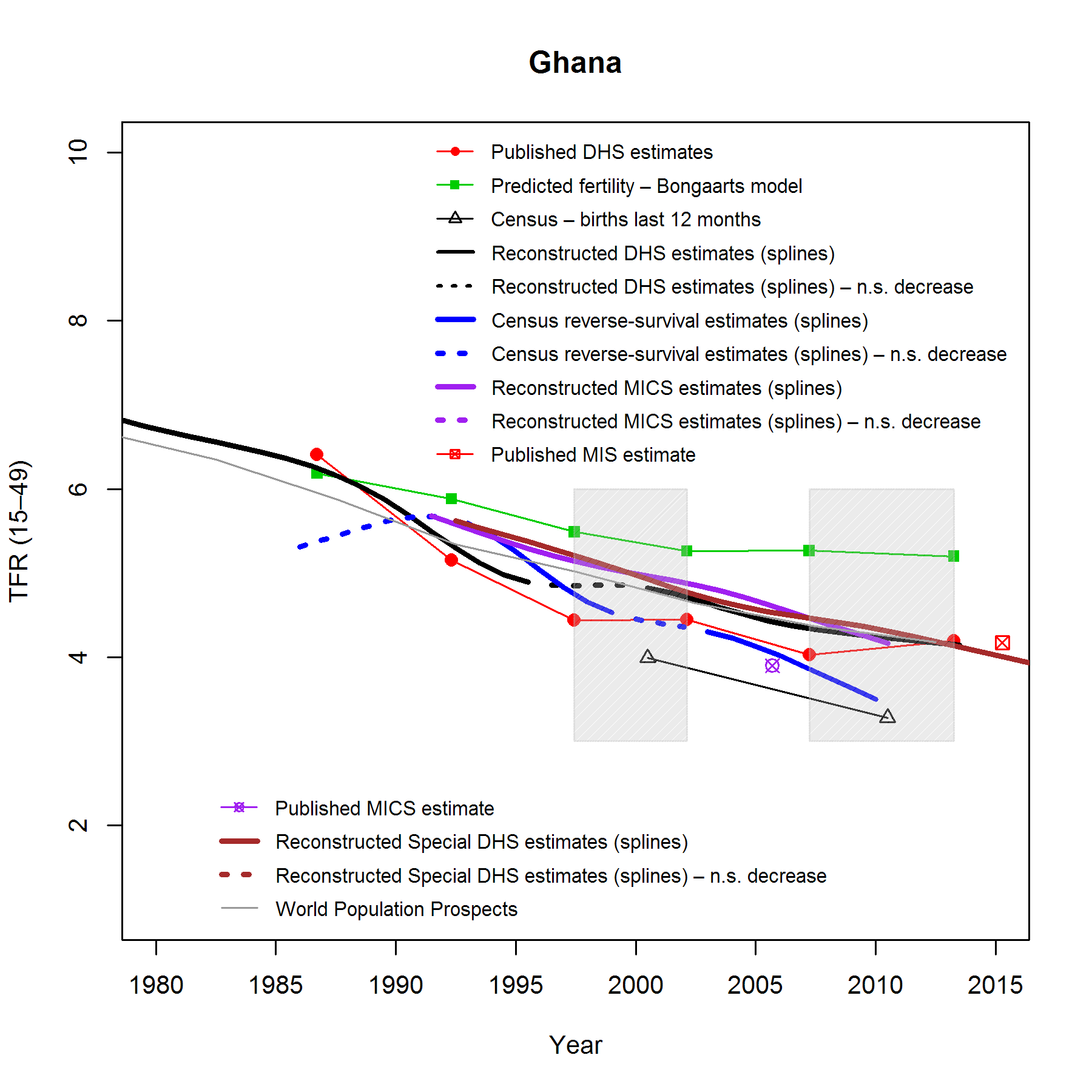 |
| 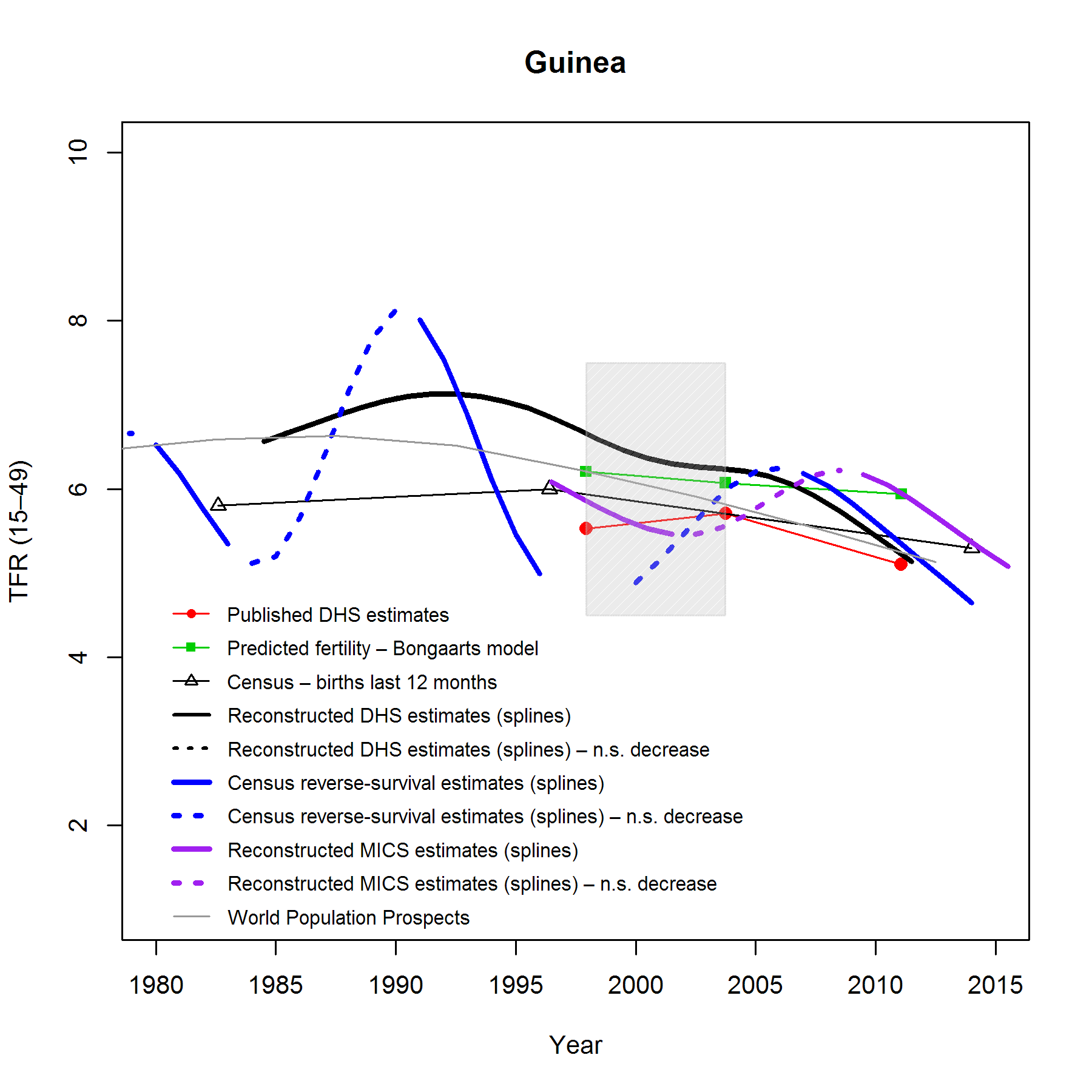 | 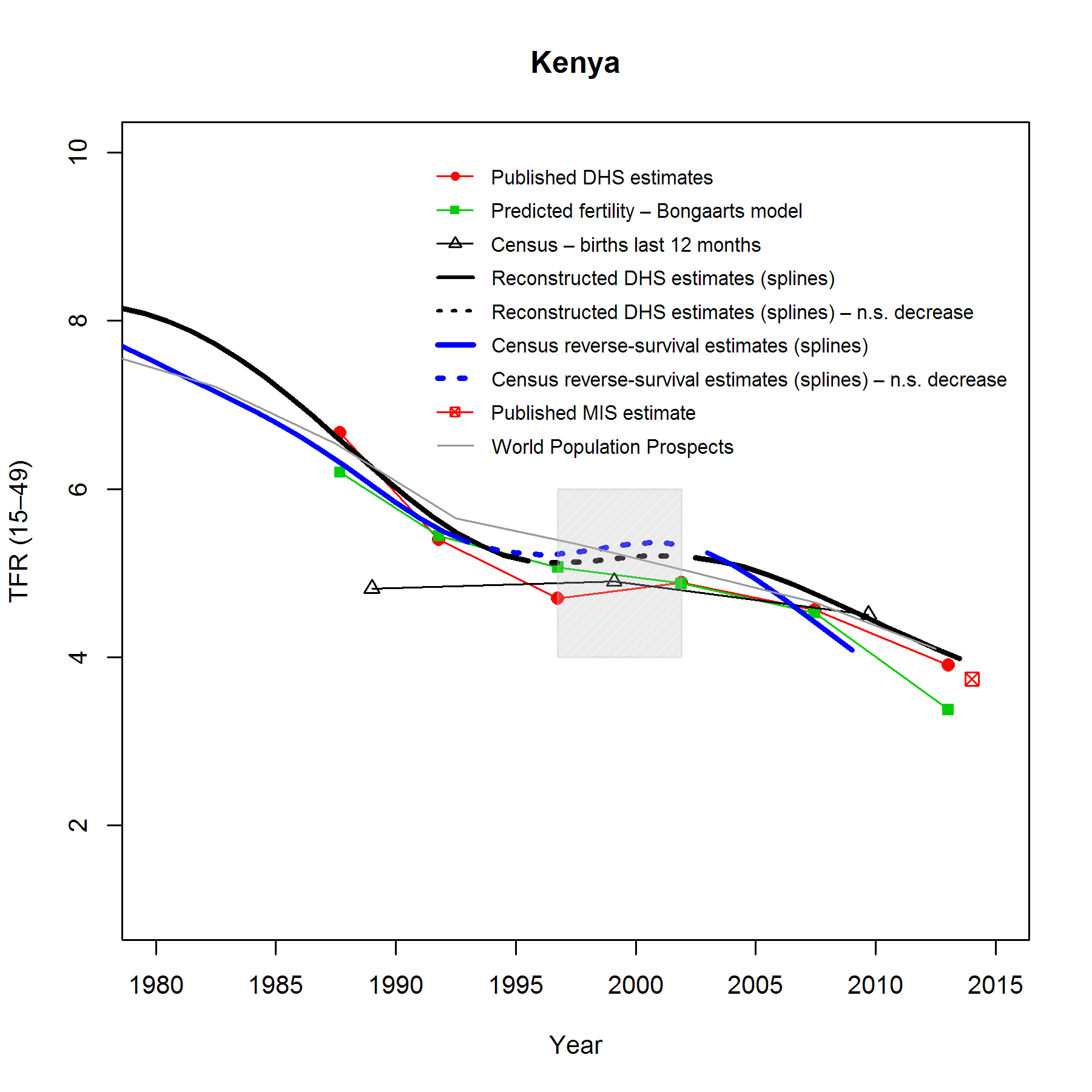 |
| 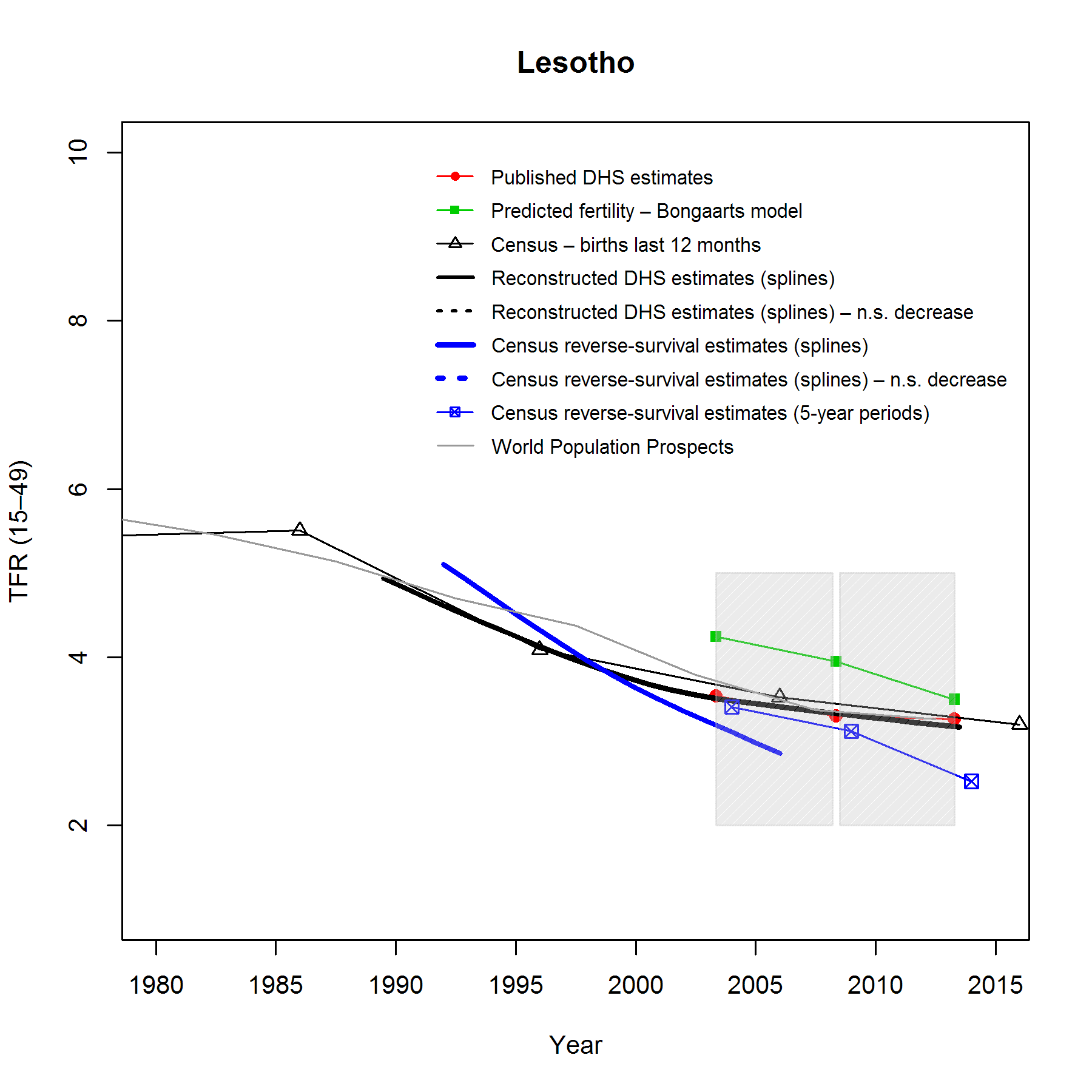 | 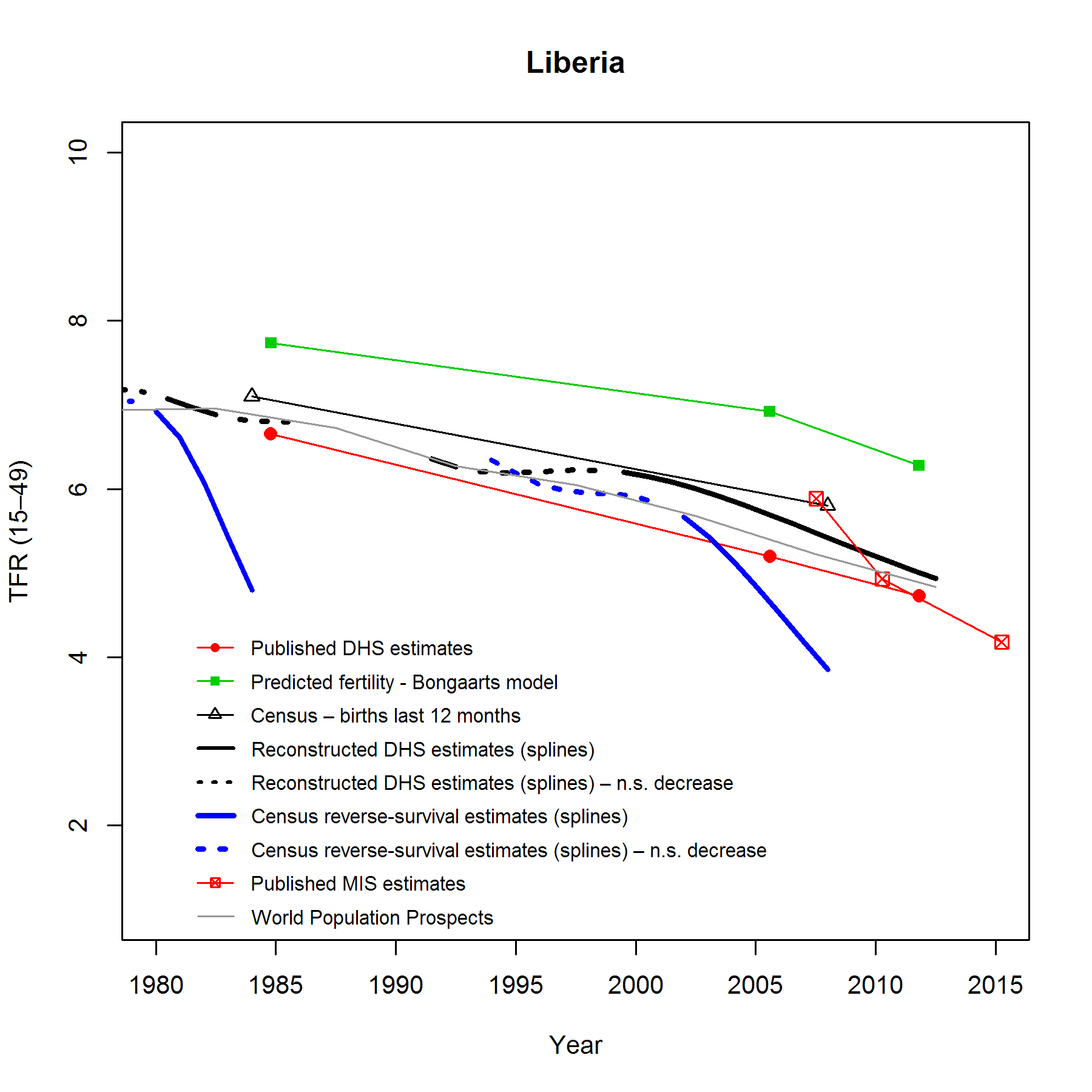 |
| 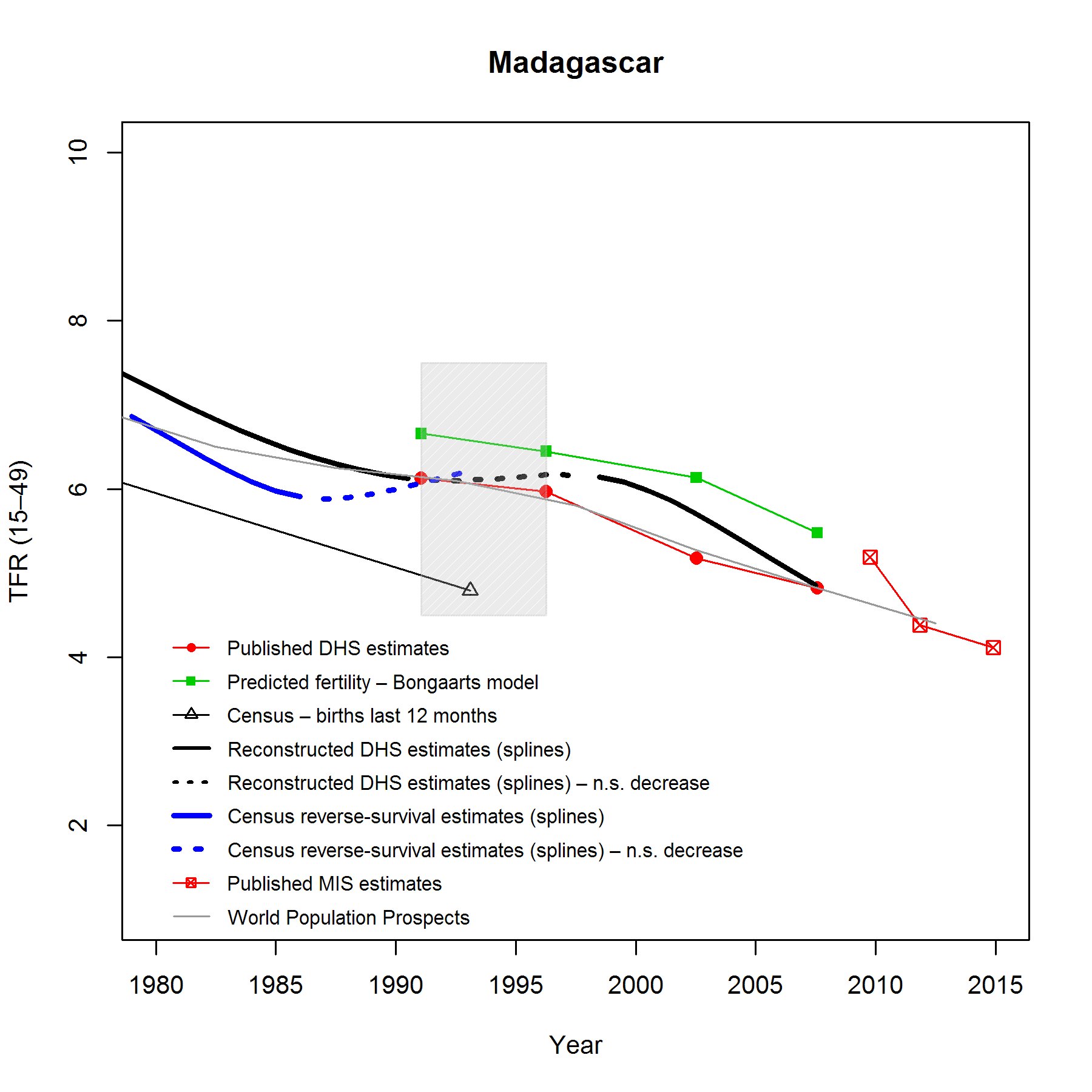 | 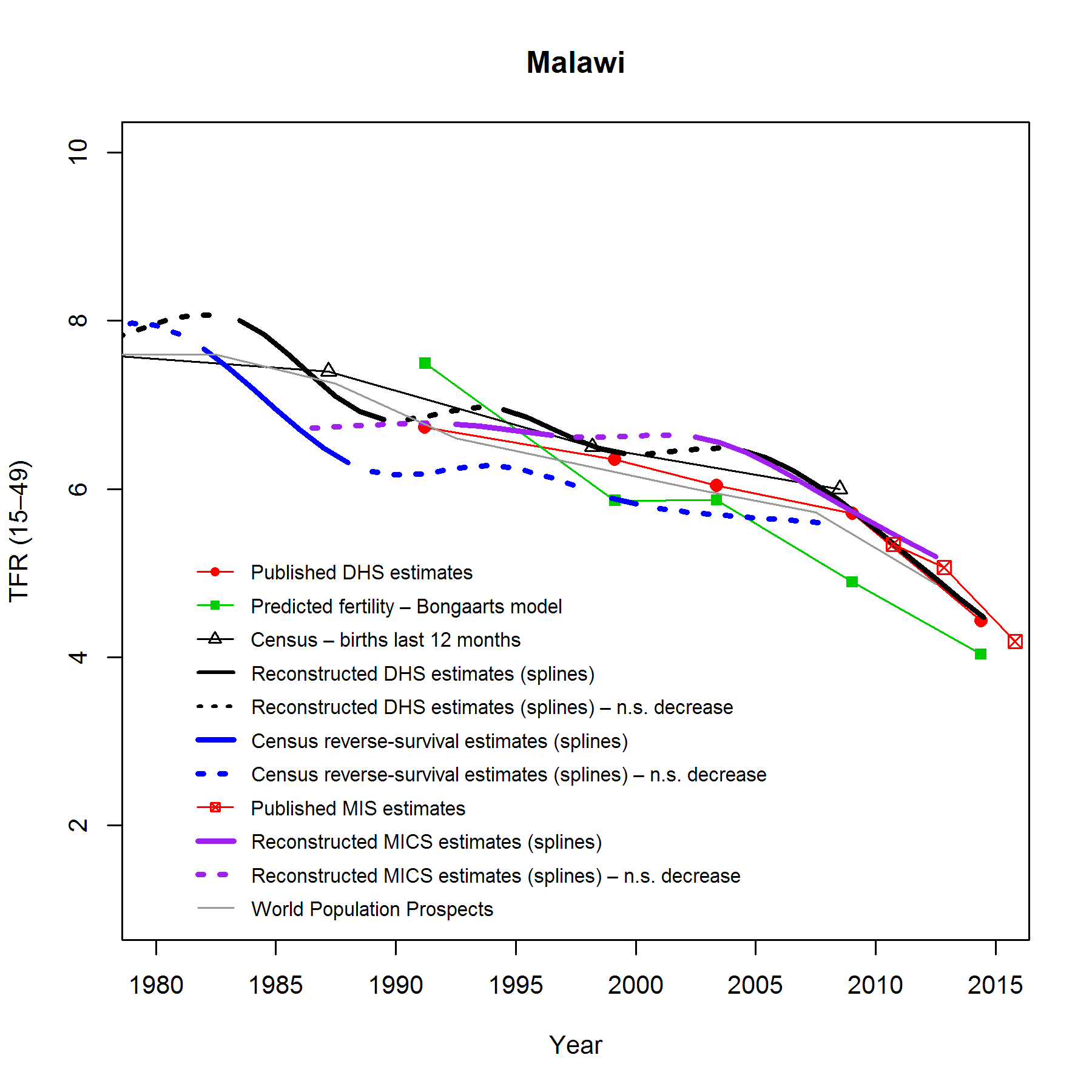 |
| 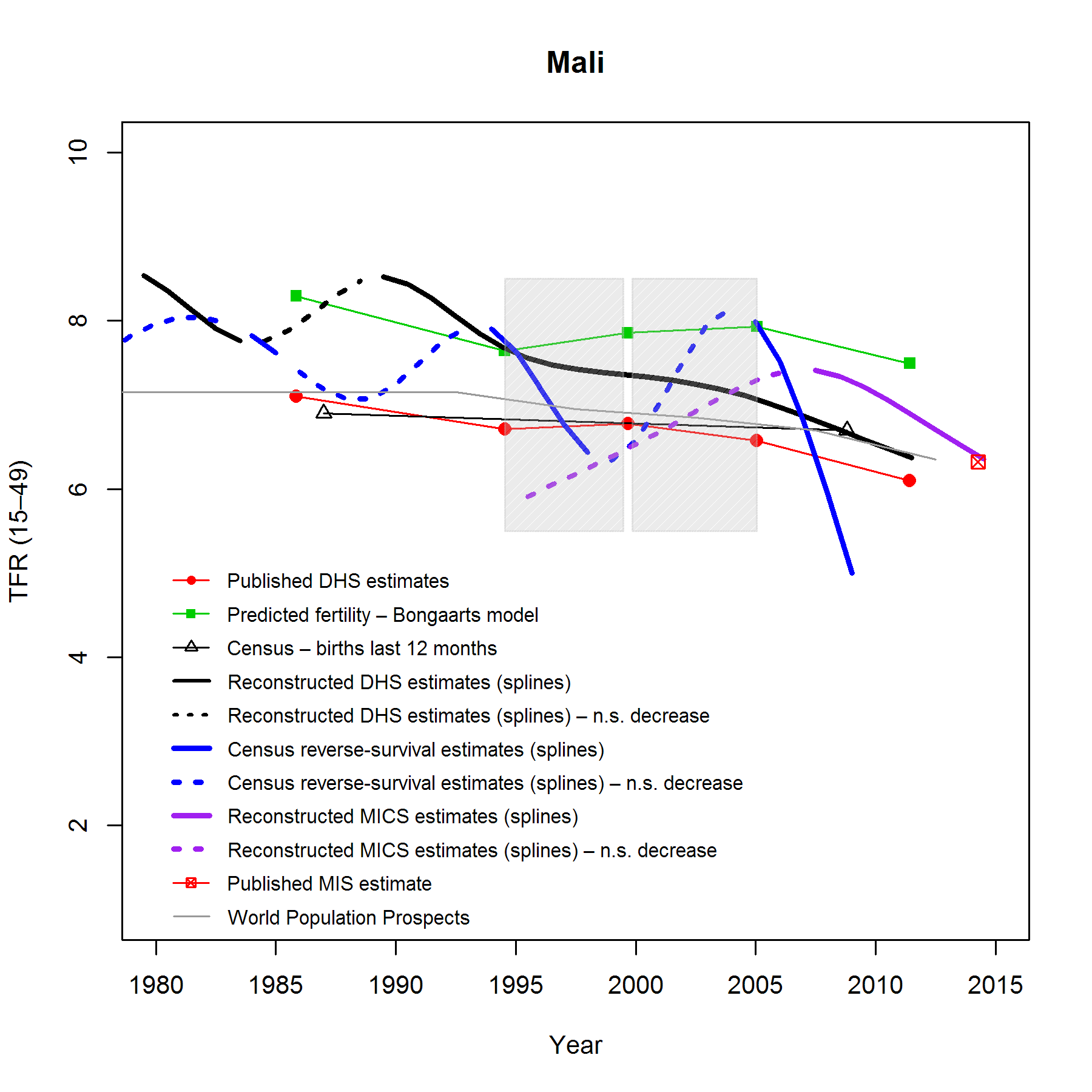 | 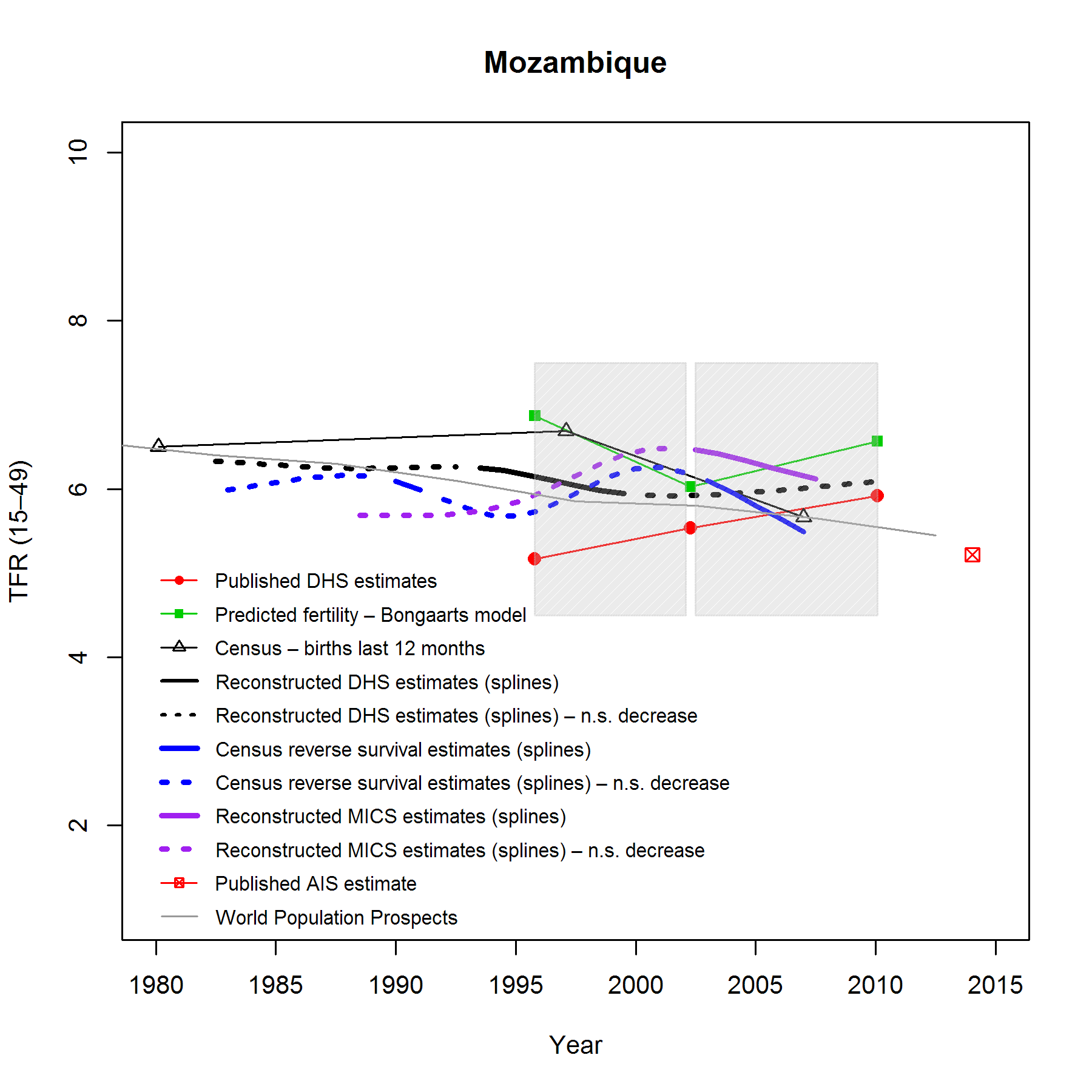 |
| 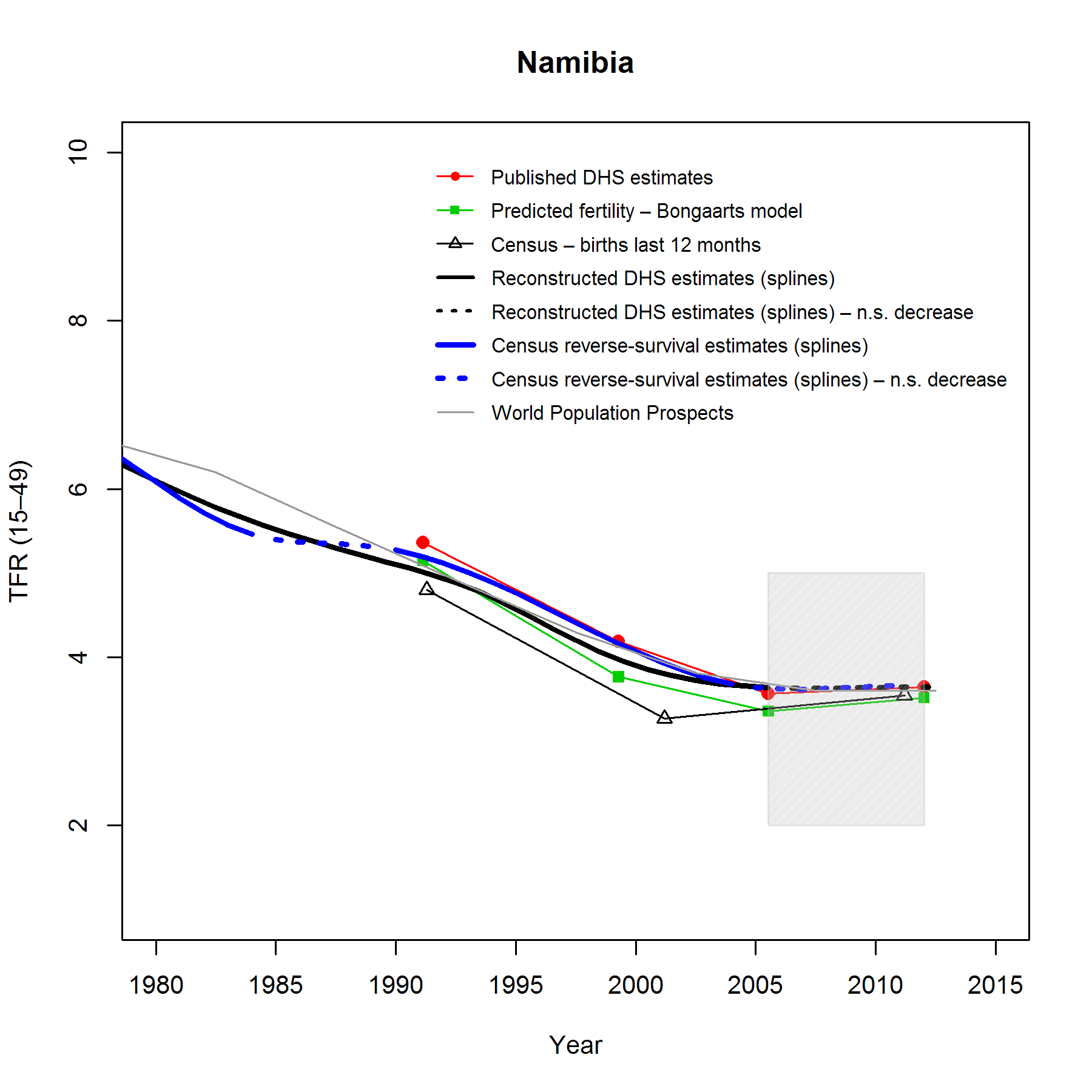 | 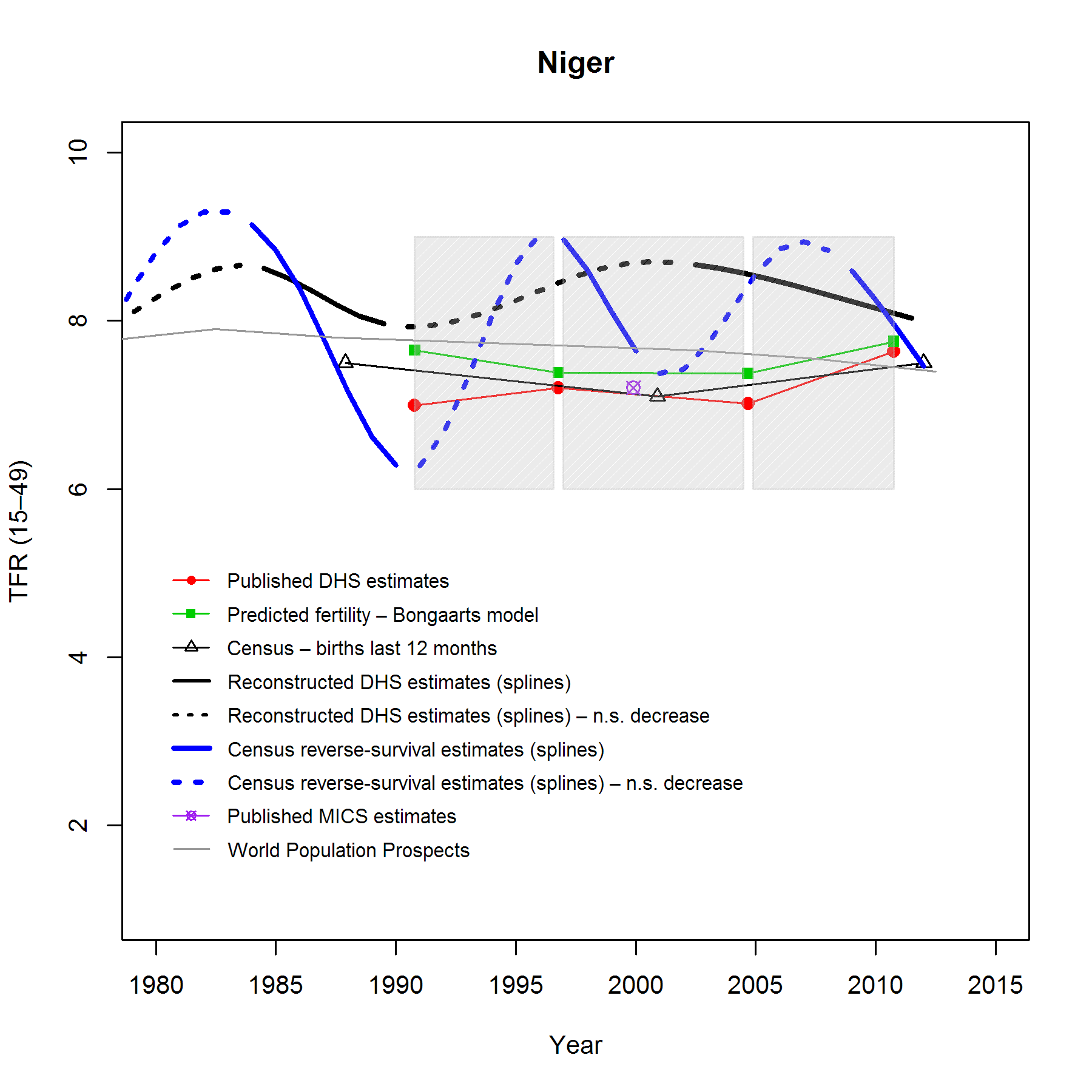 |
| 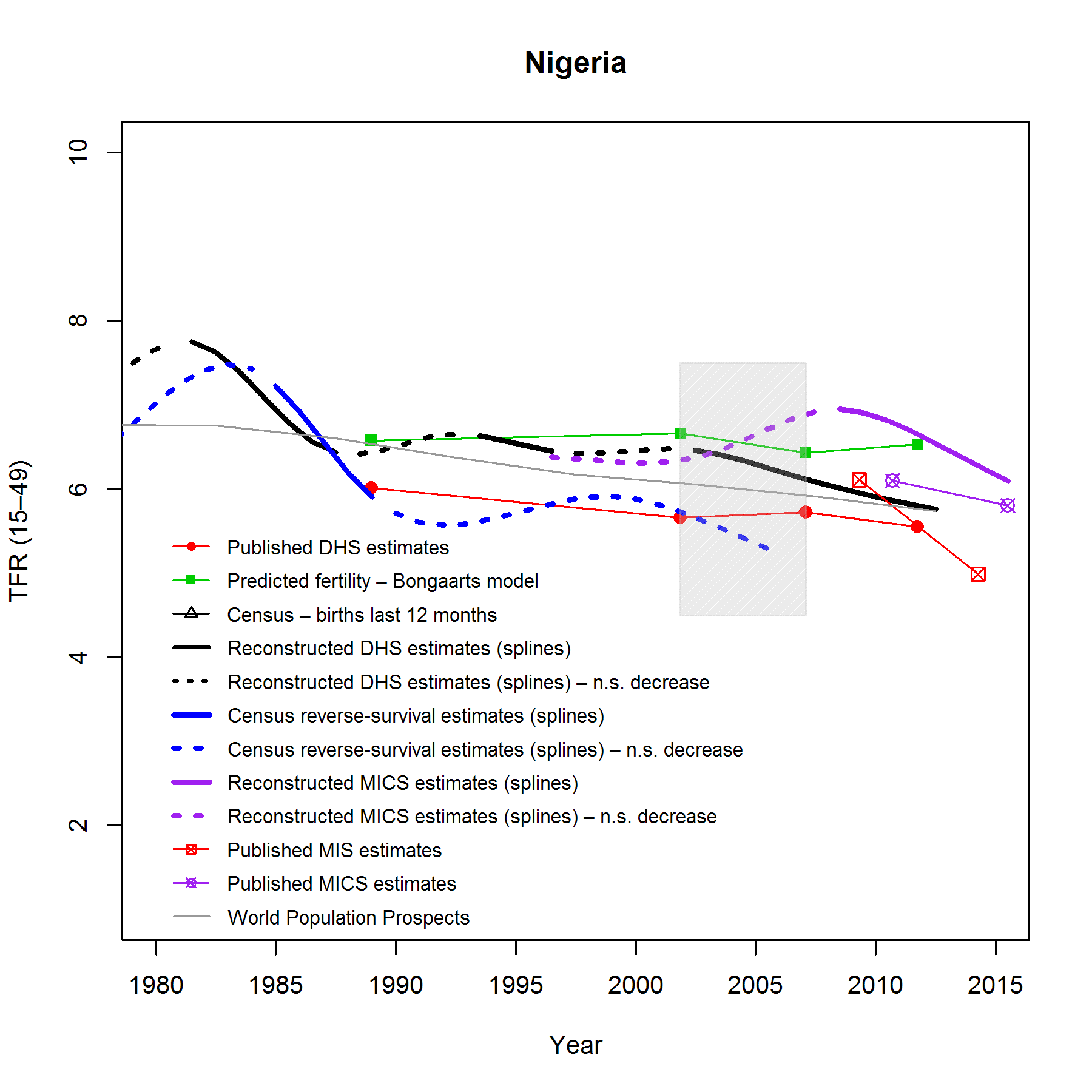 | 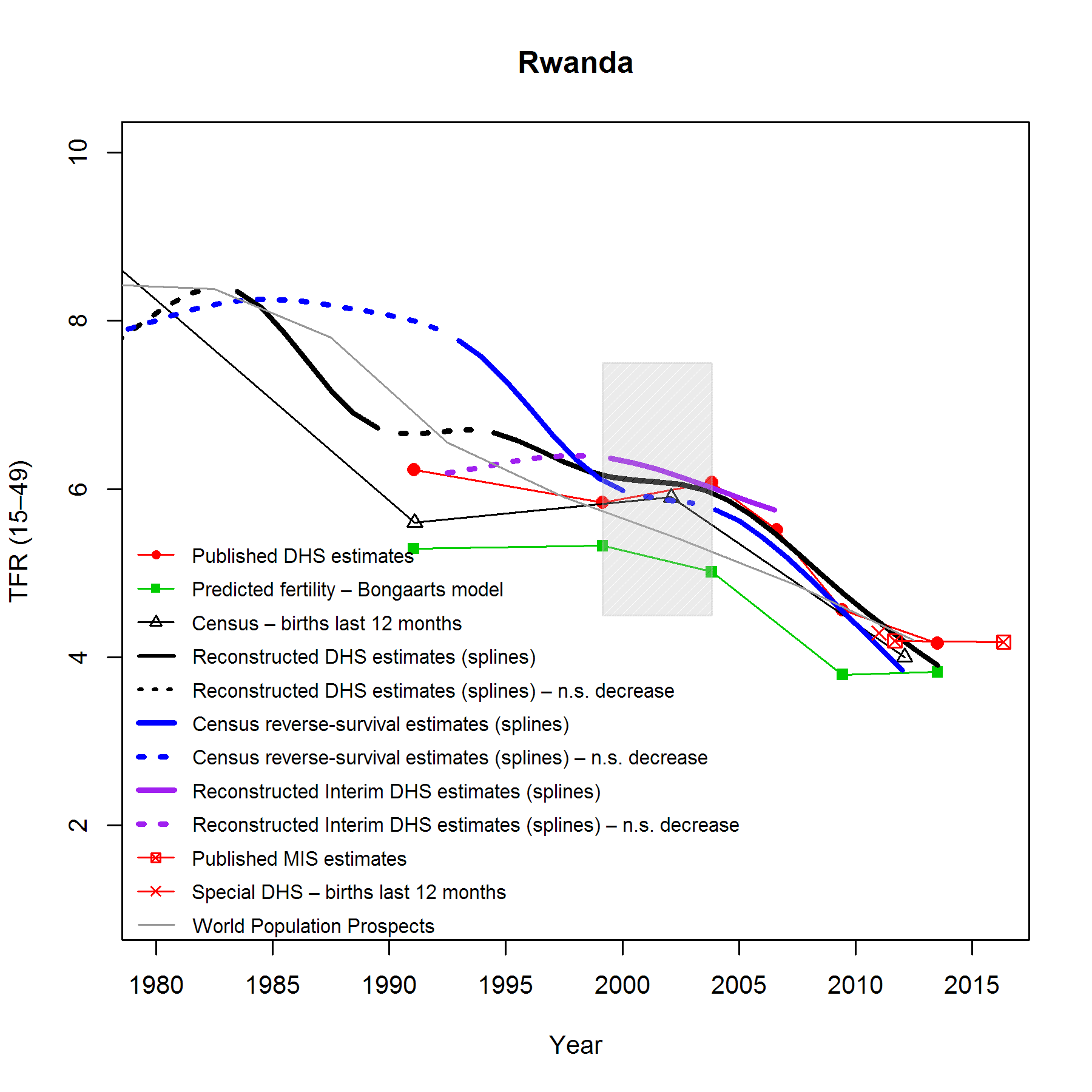 |
| 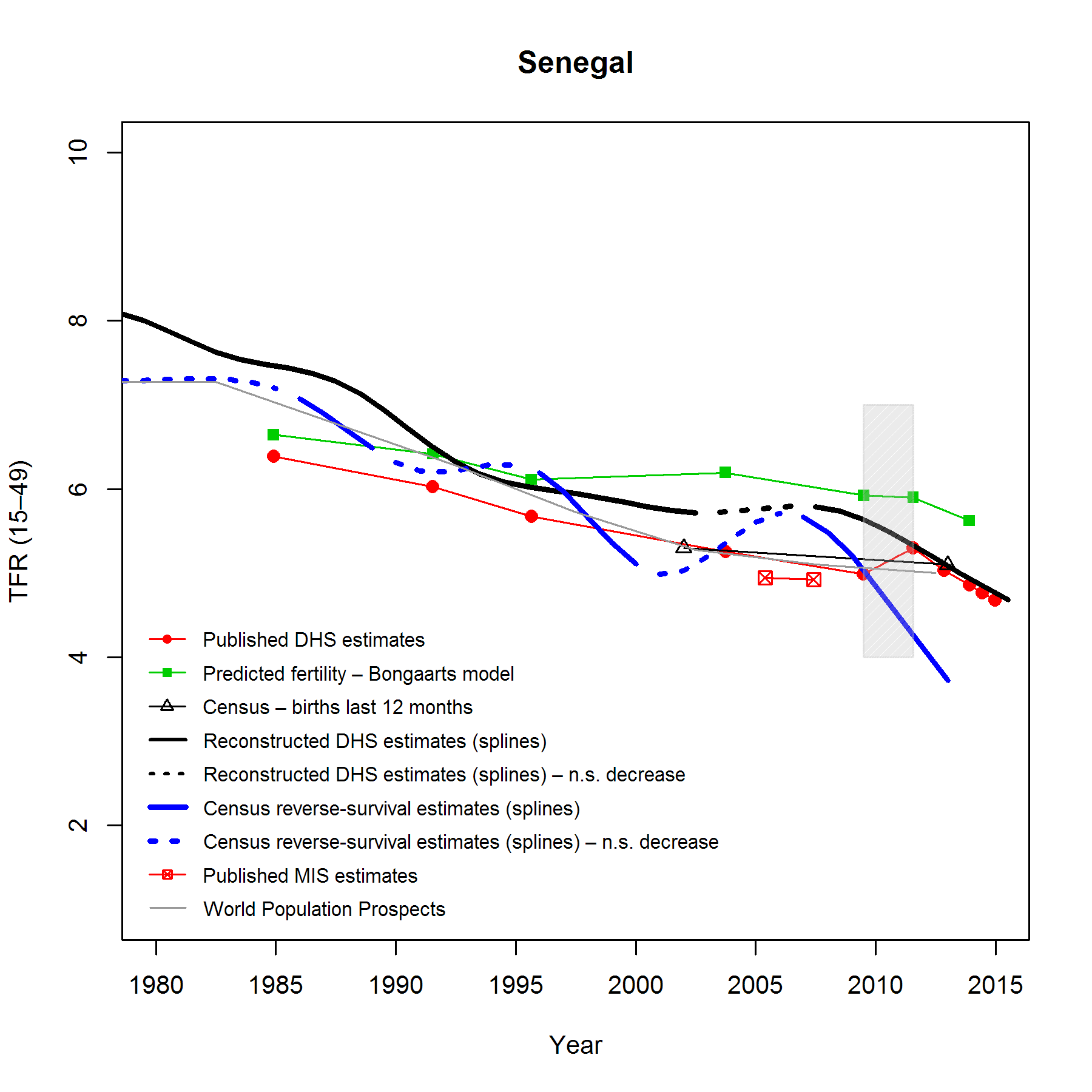 | 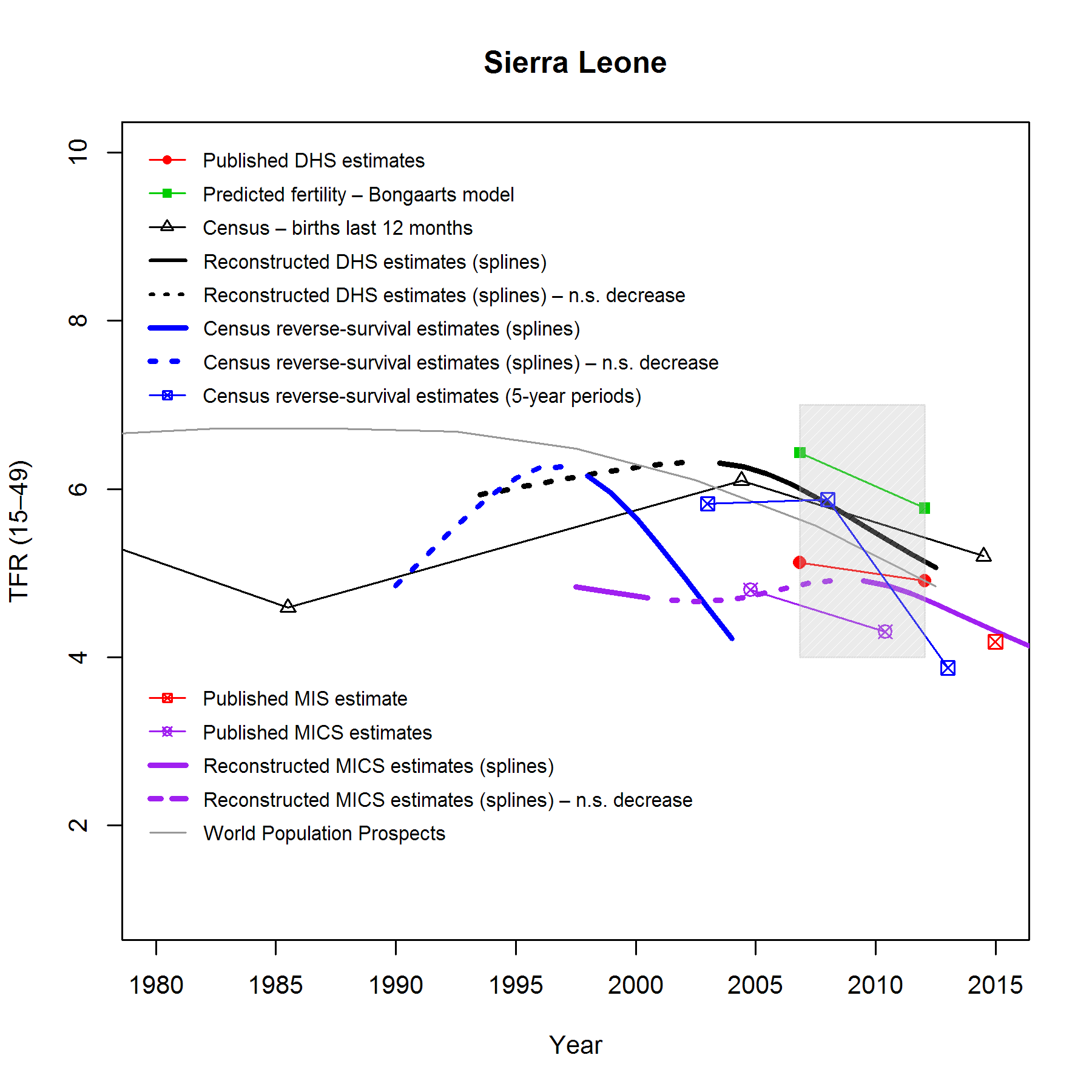 |
| 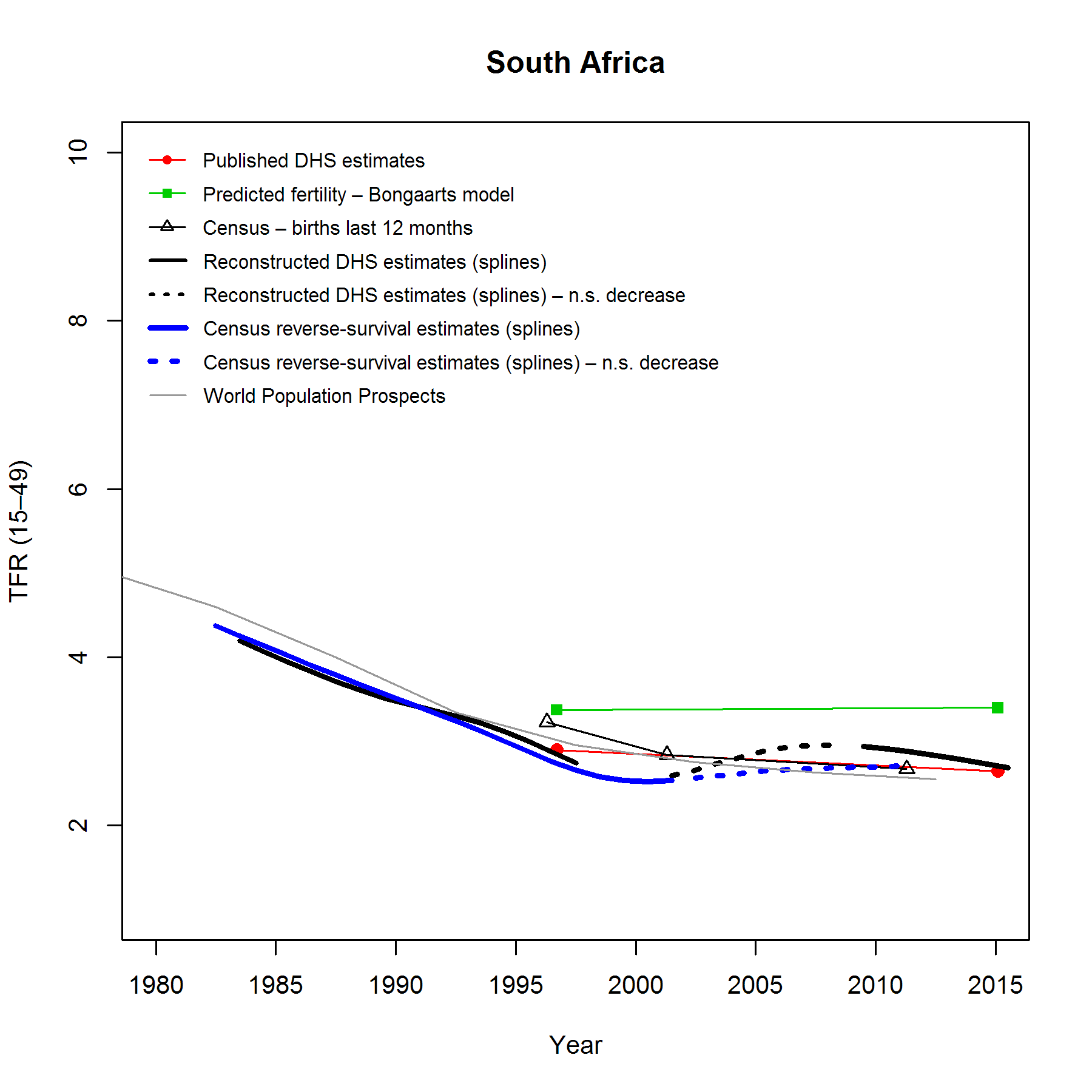 | 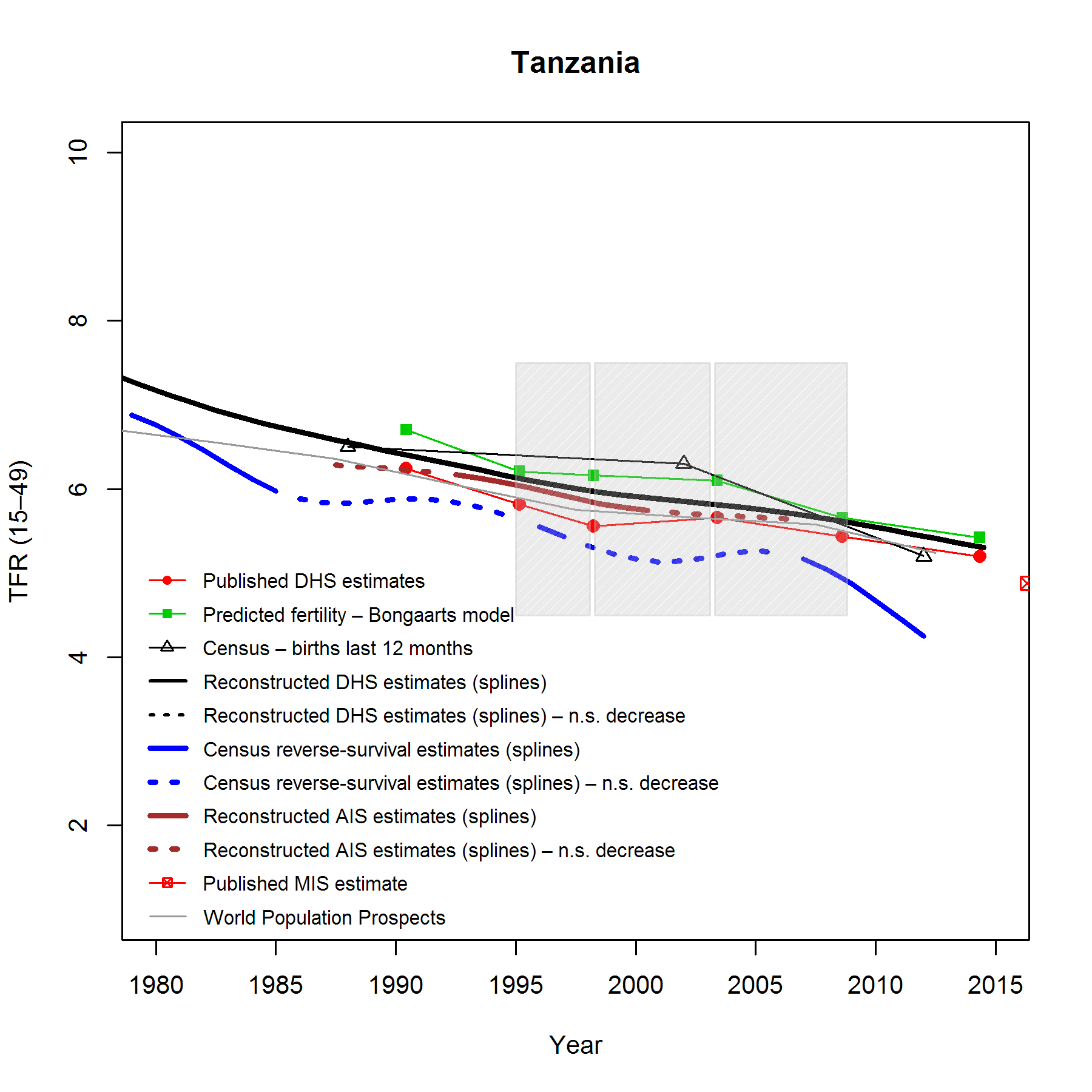 |
| 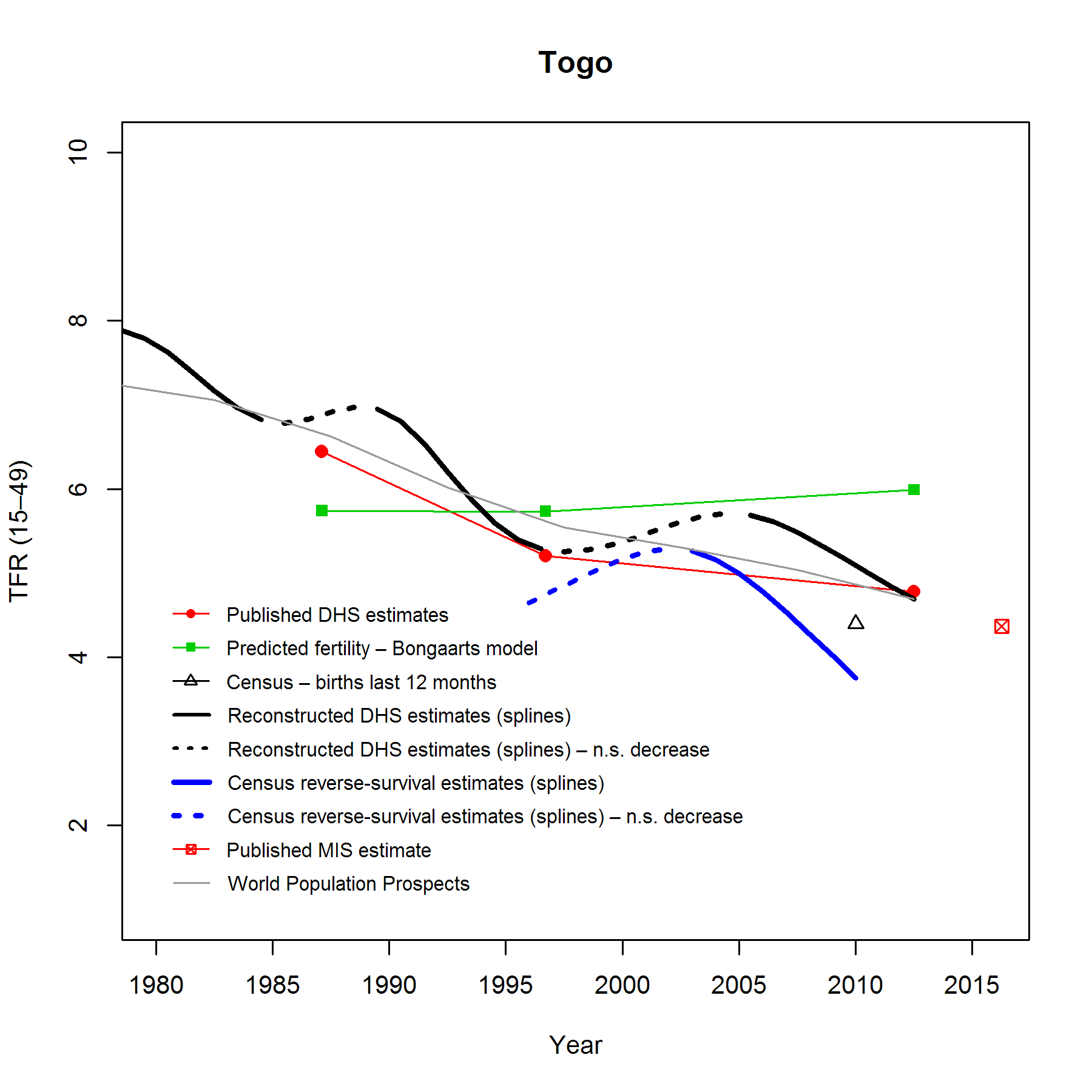 | 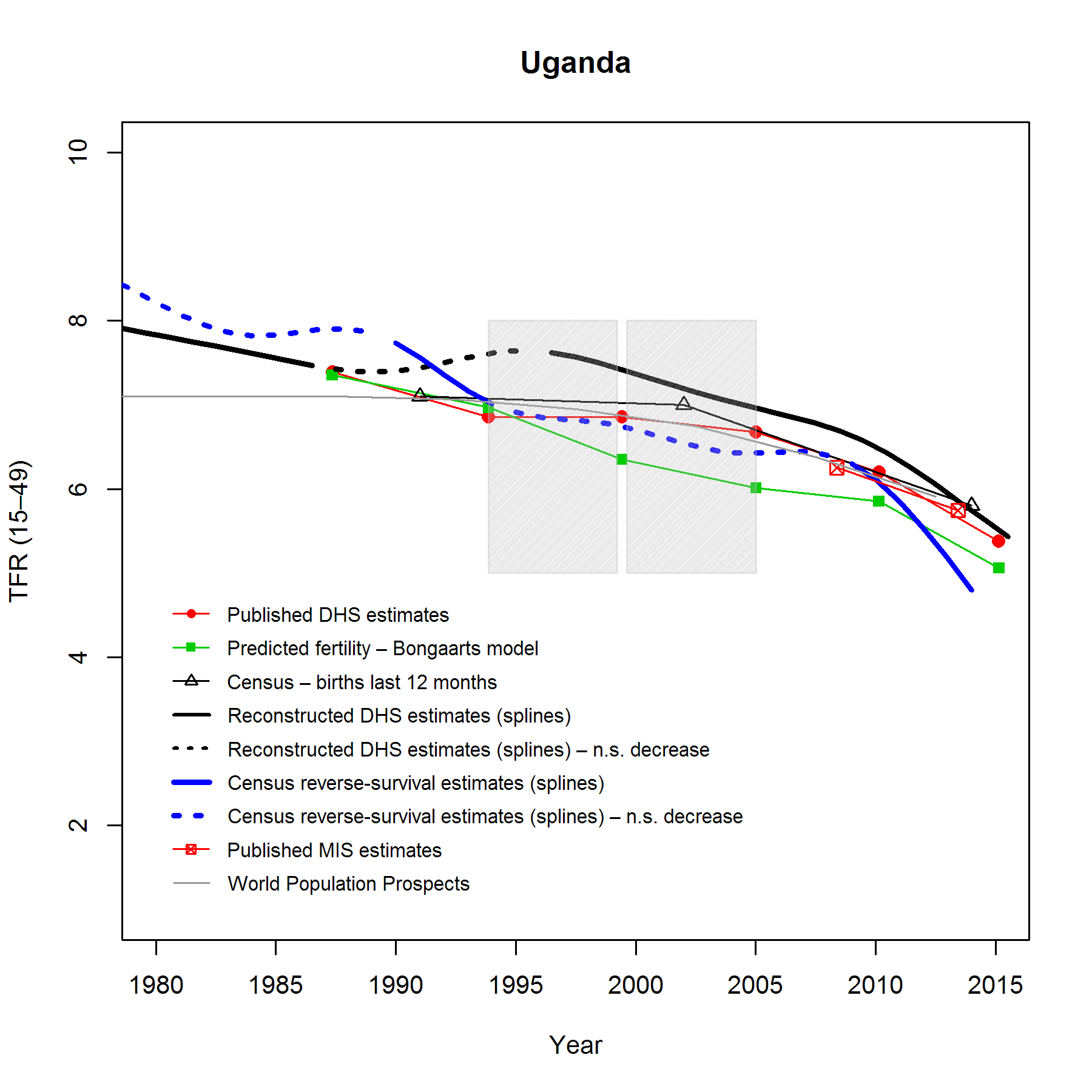 |
| 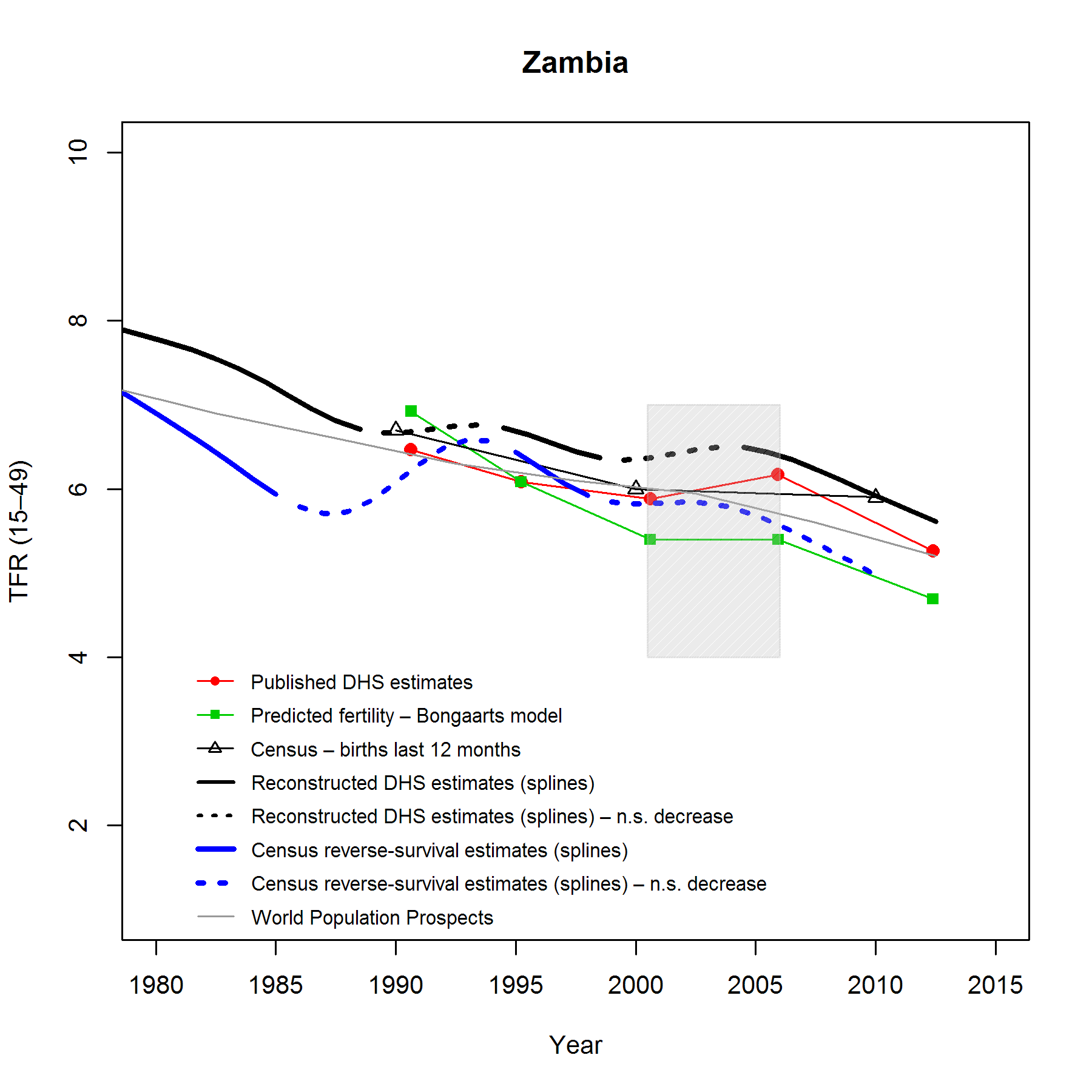 | 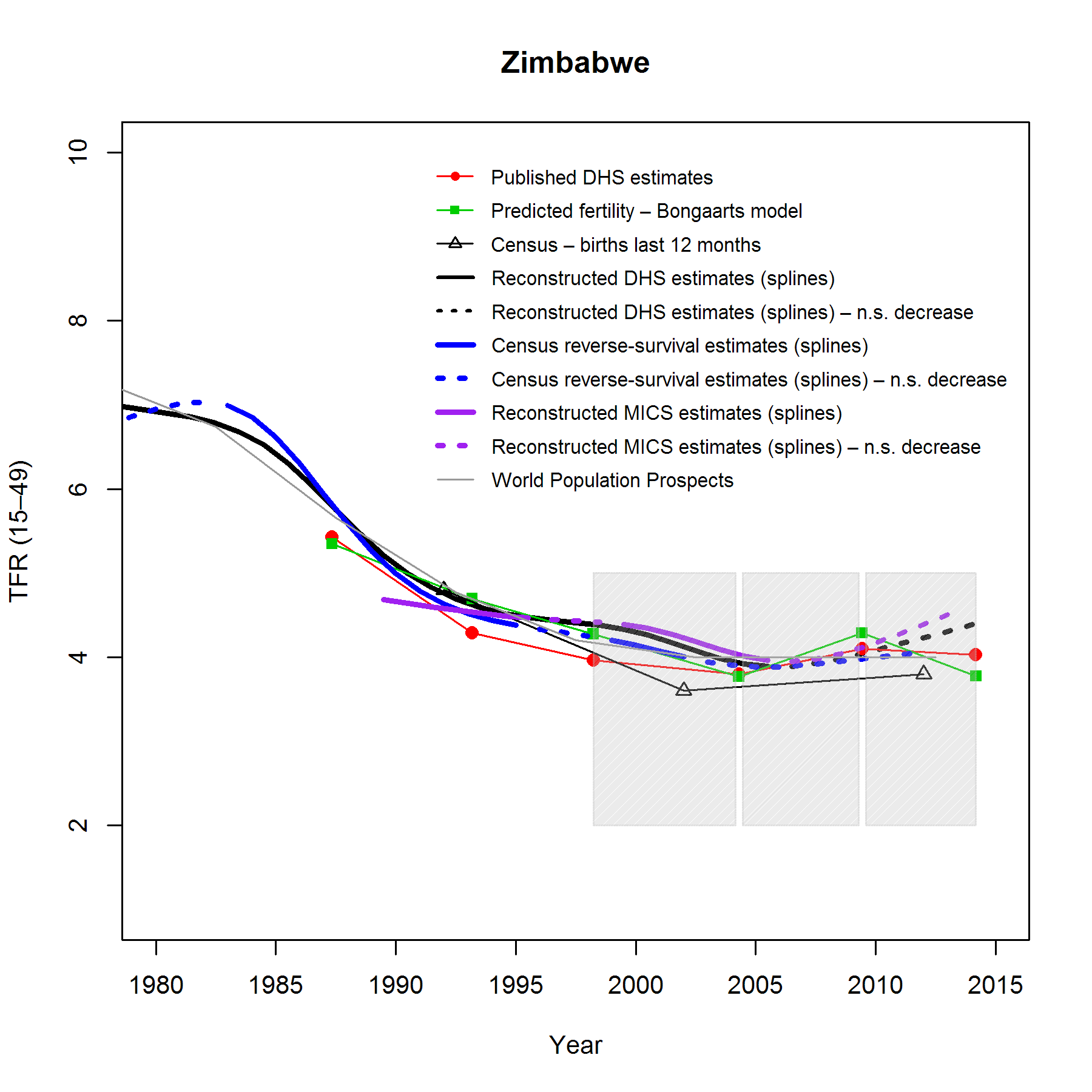 |
| NOTE: Grey shaded areas represent stalls (nonsignificant declines) identified by comparing published total fertility rates (3 years preceding the survey) from succesive DHS.  N.S. decrease = nonsignificant decrease (p>0.10). | |
